# Integrative analysis of transcriptome and metabolome reveals flavonoid biosynthesis regulation in *Rhododendron pulchrum* petals

**DOI:** 10.1186/s12870-022-03762-y

**Published:** 2022-08-16

**Authors:** Xi Xia, Rui Gong, Chunying Zhang

**Affiliations:** Shanghai Urban Plant Resources Development and Application Engineering Research Center, Shanghai Botanical Garden, Shanghai, China

**Keywords:** *Rhododendron pulchrum*, Metabolome, Transcriptome, Flavonoid biosynthesis

## Abstract

**Background:**

Color is the major ornamental feature of the *Rhododendron* genus, and it is related to the contents of flavonoid in petals. However, the regulatory mechanism of flavonoid biosynthesis in *Rhododendron pulchrum* remains unknown. The transcriptome and metabolome analysis of *Rhododendron pulchrum* with white, pink and purple color in this study aimed to reveal the mechanism of flavonoid biosynthesis and to provide insight for improving the petal color.

**Results:**

Flavonoids and flavonols are the major components of flavonoid metabolites in *R.pulchrum*, such as laricitrin, apigenin, tricin, luteolin, isoorientin, isoscutellarein, diosmetin and their glycosides derivatives. With transcriptome and metabolome analysis, we found *CHS, FLS, F3’H, F3′5’H, DFR, ANS*, *GT, FNS*, *IFR* and *FAOMT* genes showed significantly differential expression in cultivar ‘Zihe'. *FNS and IFR* were discovered to be associated with coloration in *R.pulchrum* for the first time. The *FNS* gene existed in the form of *FNSI.* The *IFR* gene and its related metabolites of medicarpin derivatives were highly expressed in purple petal. In cultivar ‘Fenhe', up-regulation of *F3’H* and *F3′5’H* and down-regulation of *4CL, DFR, ANS,* and *GT* were associated with pink coloration. With the transcription factor analysis, a subfamily of *DREBs* was found to be specifically enriched in pink petals. This suggested that the *DREB* family play an important role in pink coloration. In cultivars ‘Baihe', flavonoid biosynthesis was inhibited by low expression of *CHS*, while pigment accumulation was inhibited by low expression of *F3′5'H, DFR*, and *GT*, which led to a white coloration.

**Conclusions:**

By analyzing the transcriptome and metabolome of *R.pulchrum*, principal differential expression genes and metabolites of flavonoid biosynthesis pathway were identified. Many novel metabolites, genes, and transcription factors associated with coloration have been discovered. To reveal the mechanism of the coloration of different petals, a model of the flavonoid biosynthesis pathway of *R.pulchrum* was constructed. These results provide in depth information regarding the coloration of the petals and the flavonoid metabolism of *R.pulcherum*. The study of transcriptome and metabolome profiling gains insight for further genetic improvement in *Rhododendron*.

**Supplementary Information:**

The online version contains supplementary material available at 10.1186/s12870-022-03762-y.

## Background

Floral color is a main ornamental feature of plants. Several factors, including Vacuole pH, metal ions, pigment composition, and the structure of the petal, influence flower color [1]. One of the most critical factors is the pigment composition, which contains flavonoids, carotenoids, betalains, and chlorophylls [2]. Flavonoids produce a variety of colors ranging from pale yellow to deep blue [3], which have been divided into flavonoids, flavonols, anthocyanins, isoflavones, flavan-3-ols, dihydroflavonols, dihydroflavones, and chalcons [4, 5]. Among these, chalcones were reported in deep yellow color, while flavones and flavonols in faintly yellow or almost colorless [6]. A variety of colors can be produced by anthocyanins [7]. To date, nearly 700 anthocyanin derivatives have been identified in plants [8].

The flavonoid biosynthesis is a complex process in which structural genes such as early and late biosynthetic genes (EBGs and LBGs) are involved [9]. These EBGs encode enzymes that provide precursors for flavonoid synthesis, including phenylalanine ammonia-lyase (*PAL*), cinnamate-4-hydroxylase (*C4H*), 4-coumarate CoA ligase 4 (*4CL*), chalcone synthase (*CHS*), and chalcone isomerase (*CHI*). LBGs are responsible for the synthesis of proanthocyanidins, which includes flavanone 3-hydroxylase(*F3H*), flavonoid 3’-hydroxylase(*F3’H*), flavonoid 3’,5’-hydroxylase(*F3′5’H*), dihydroflflavonol 4-reductase (*DFR*)*, *anthocyanidin synthase (*ANS*), flavonol synthase (*FLS*), glucosyltransferase (*GT*), etc. It was reported *CHS* had the potential to affect petal coloration [10, 11]. A high expression of *CHS, DFR,* and *ANS* genes led to an accumulation of anthocyanins [12], while the insertion of *ANS* coding led to a reduction in anthocyanin content [13]. Yang et al. observed that the expression of *CHI, C3 'H* and *F3 'H* genes increased in red *Eucommia ulmoides* leaves, while the expression of *F3 ′5'H* and *GT* genes decreased [14]. In addition, flavonoid biosynthesis is also jointly controlled by a variety of transcription factors, including *MYB, bHLH, TTG1* (*WD40* repeat protein), *TTG2* (*WRKY44*), *TT16/AGL32* (*MADS* protein) and *TT1* [15]. In recent years, genes and transcription factors involved in anthocyanin biosynthesis have been identified in many plants [16–20].

The genus *Rhododendron* contains brightly colored flowers and is of great use in horticulture [21]. Flavanols and anthocyanins are the main pigments in *Rhododendron* [22], their quantities determine the petal color ranging from light pink to violet [23–28]. At present, several studies have been done on genes and transcription factors involved in the flavonoid biosynthesis pathway in *Rhododendron* [29–31]. *Rhododendron pulchrum* Sweet is a species of *Rhododendron* belonging to the Ericaceae family and is widely cultivated in temperate Europe, Asia and North America [32]. It is highly resistant and widely cultivated in the Yangtze Delta region [33]. Based on the study of Qian et al., the main anthocyanin in purple petals is Peonidin, while Pelargonidin is the main component in pink petals, and no anthocyanin is detected in white petals of *R.pulchrum* [34]. There were 149 flavonoids and their derivatives identified in *R.pulchrum* cultivars with different colors, among them flavone and C-glycosylated flavone were the most important flavonoid metabolites [35]. In one transcriptome analysis of *R.pulchrum* petals, it was found that most differentially expressed genes (DEGs) were located between flower buds and early flowering, while transcripts for the genes *MYC2, TIR1, CYCD3, COL-1*, and *EIN3* peaked at flower buds [36]. However, the regulatory mechanism of flavonoid biosynthesis in *R.pulchrum* remains unclear.

Recent studies have used integrated multi-omics analyses to identify and analyze traits related genes and pathways in plants [5, 6, 37–41]. A combined analysis of transcriptomes and metabolomes can directly and accurately reflect organismal change. We performed the analysis of metabolome and transcriptome to understand the flavonoid biosynthesis pathway in petals of *R.pulchrum* for the first time. The findings of this study explain the coloring mechanism and provide a theoretical basis for color breeding of *R.pulchrum*.

## Results

### Six hundred eighty-eight of differential metabolites were identified in three cultivars of *R.pulchrum*

**Fig. 1:**
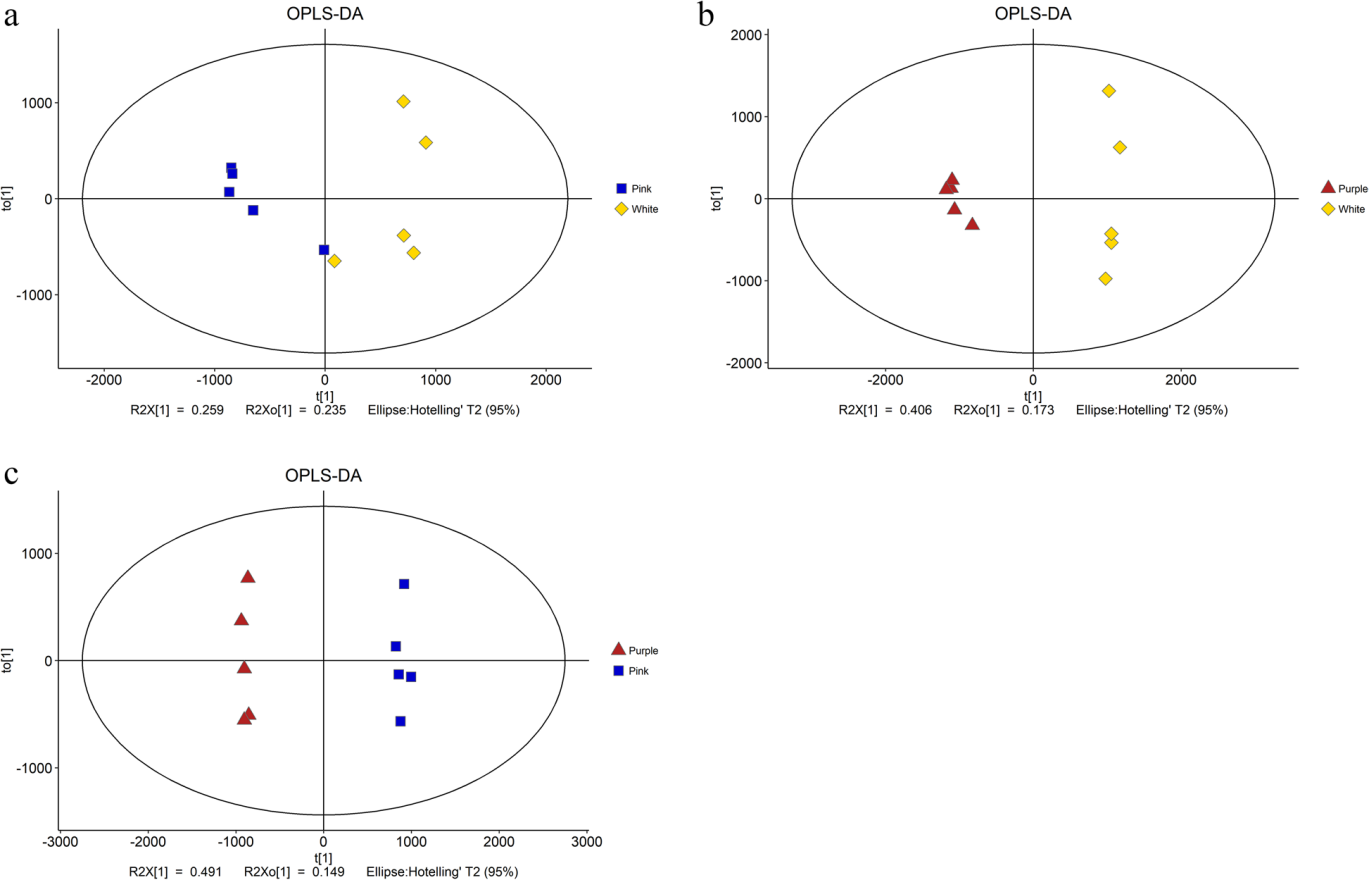
OPLS-DA score plot of three materials and numbers of potential markers for each color petals. Note: **a** OPLS-DA score plot of ‘Baihe’ and ‘Fenhe’ petals; **b** OPLS-DA score plot of ‘Zihe’ and ‘Baihe’ petals; **c** OPLS-DA score plot of ‘Fenhe’ and ‘Zihe’ petals

Liquid chromatography–mass spectrometry (LC–MS) analysis was conducted to identify the compositions of metabolites within cultivars 'Baihe', 'Fenhe' and 'Zihe' petals. According to the results of the orthogonal projections to latent structures discriminant analysis (OPLS-DA), there is a clear separation of three colors, 'Baihe' and 'Fenhe' show a closer relationship when compared with 'Zihe' (Fig. 1). In all, 688 differential metabolites were identified in three comparison groups (VIP ≥ 1 and *P* < 0. 05). 176 differential metabolites were found between cultivars 'Baihe' and ‘Fenhe’. Of these, 109 were up-regulated, and 67 were down-regulated in ‘Fenhe’. There were 252 (139 up-regulated and 113 down-regulated) and 260 (155 up-regulated and 105 down-regulated) differential metabolites in cultivar 'Zihe' compared to cultivars 'Baihe' and 'Fenhe', respectively (Fig. S1).

### One hundred one flavonoid-related differential metabolites were discovered in three cultivars of *R. pulchrum*

**Fig. 2:**
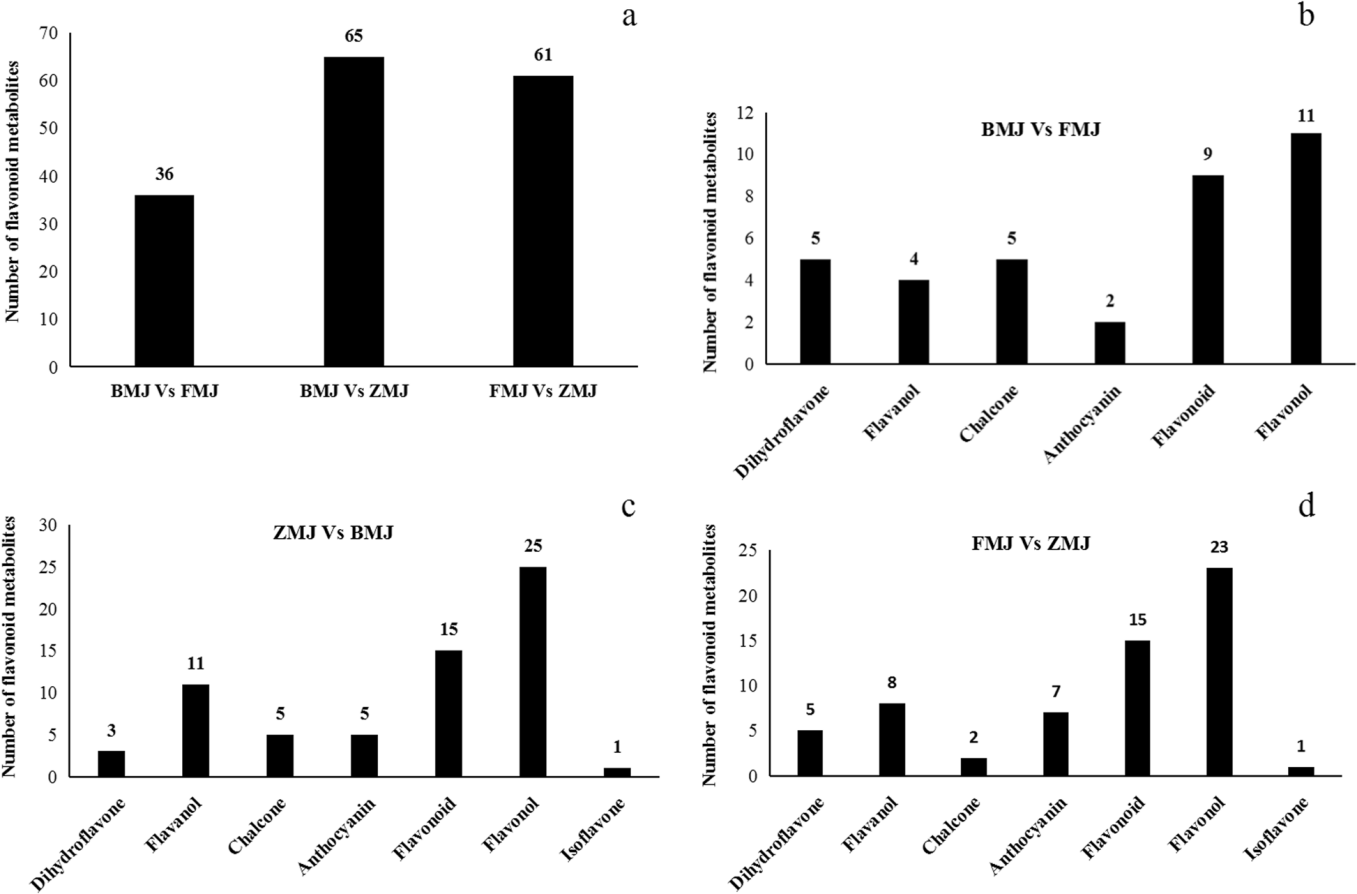
Flavonoid metabolites in *R.pulchrum* with different colors. Note: BMJ, cultivar ‘Baihe’; FMJ, cultivar ‘Fenhe’; ZMJ, cultivar ‘Zihe’. **a** Measurement of total flavonoids in *R.pulchrum* with different colors. **b** Flavonoid metabolites in ‘Baihe’ and ‘Fenhe’. **c** Flavonoid metabolites in ‘Zihe’ and ‘Baihe’. **d** Flavonoid metabolites in ‘Fenhe’ and ‘Zihe’

**Fig. 3:**
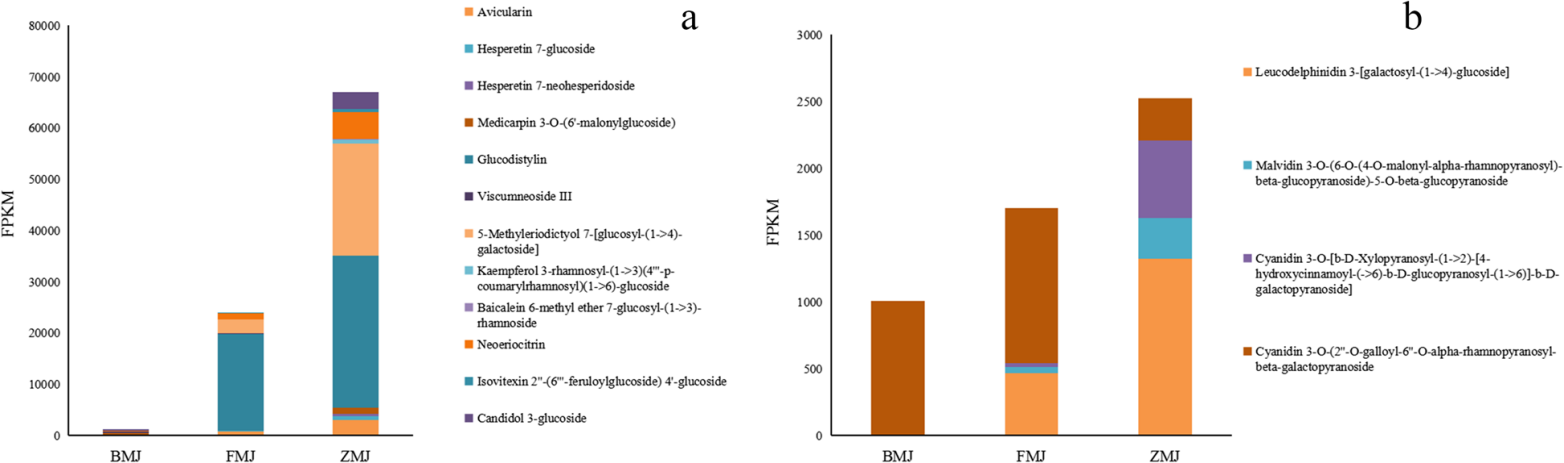
Fold change of the flavonoid and anthocyanin metabolites in *R.pulchrum* with different colors. Note: BMJ, cultivar ‘Baihe’; FMJ, cultivar ‘Fenhe’; ZMJ, cultivar ‘Zihe’. **a** Flod change of the flavonoid metabolites. **b** Fold change of the anthocyanin metabolites

To understand the coloration mechanism in more detail, our study focused on the metabolite components enriched in the flavonoid biosynthesis pathway. 36 flavonoid related metabolites were identified in cultivars of 'Baihe' (BMJ) and 'Fenhe' (FMJ) group, 65 in cultivars 'Zihe' (ZMJ) and 'Baihe' (BMJ) group, and 61 in cultivars 'Fenhe' (FMJ) and 'Zihe' (ZMJ) group (Fig. 2a). The patterns of metabolites accumulation between different colors were studied by hierarchical cluster analysis (HCA) (Fig. S2). For identifying the differential expression metabolites, we defined the criterion of the absolute Log_2_FC ≥ 1 and VIP value ≥ 1. According to the flavonoid structure of cultivars 'Baihe' and 'Fenhe' group, we compared the expression levels of 36 metabolites including five dihydroflavones, four flavanols, five chalcones, two anthocyanidin, nine flavonoids and 11 flavonols (Fig. 2b). In them, 13 metabolites were up-regulated and five down-regulated in cultivars ‘Fenhe’. Neoeriocitrin, Hesperetin 7-glucoside, Avicularin, Viscumneoside III, and Glucodistylin (Log_2_ FC = 2.67–3.73) are higher in cultivar ‘Fenhe’ than in ‘Baihe’ (Table S1), indicating that these compounds played an important role in the pink coloration of *R.pulchrum*. Three dihydroflavones, 11 flavanols, five chalcones, five anthocyanidins, 15 flavonoids, 25 flavonols, and one isoflavone were found in cultivars ‘Zihe’ and ‘Baihe’ group (Fig. 2c). 45 flavonoid metabolites with significantly differential expressions were identified (71.88% of the total), of which 29 showed up-regulated and 16 down-regulated in 'Zihe'. The amounts of Neoeriocitrin, 5-Methyleriodictyol 7-[glucosyl-(1- > 4)-galactoside], Hesperetin 7-glucoside, Kaempferol 3-rhamnosyl-(1- > 3)(4'''-p-coumarylrhamnosyl)(1- > 6)-glucoside, Hesperetin 7-neohesperidoside (Log_2_ FC = 4.87–9.69) are significantly higher in cultivar ‘Zihe’ than in ‘Baihe’ (Table S1), indicating these compounds are involved in the purple coloration of *R.pulchrum*. For cultivars 'Fenhe' and 'Zihe' group, we identified metabolites of five dihydroflavones, eight flavanols, two chalcones, seven anthocyanidins, 15 flavonoids, 23 flavonols, and one isoflavone (Fig. 2d), and 38 metabolites were differentially expressed (63.33% of the total), including 22 of them up-regulated and 16 down-regulated in cultivar ‘Zihe’. A significantly higher enrichment of Isovitexin 2''-(6'''-feruloylglucoside) 4'-glucoside, Medicarpin 3-O-(6'-malonylglucoside), Baicalein 6-methylether 7-glucosyl-(1- > 3)-rhamnoside, Kaempferol 3-rhamnosyl-(1- > 3)(4'''-p-coumarylrhamnosyl)(1- > 6)-glucoside, and Hesperetin 7-neohesperidoside(Log_2_ FC = 3.18–7.47) was found in cultivar 'Zihe' than in 'Fenhe'(Table S1). This indicates these metabolites are more specific to the purple coloration rather than pink in *R.pulchrum*. In summary, the flavonoid metabolites are primarily flavonoids and flavonols, and there are abundant amounts of Laricitrin, Apigenin, Tricin, Luteolin, Isoorientin, Isoscutellarein, Diosmetin,Triclin, and their glycosides. Importantly, it has been found that medicarpin 3-O-(6'-malonylglucoside) classified as isoflavones were more abundant in cultivar 'Zihe' than in the other two cultivars (Fig. 3a). Additionally, a total of 8 anthocyanins were identified, including cyanidin, pelargonidin, delphinidin, and malvidin. The content of malvidin 3-O-(6-O-(4-O-malonyl-alpha-rhamnopyranosyl)-beta-glucopyranoside)-5-O-beta-glucopyranoside and Cyanidin 3-O-[b-D-Xylopyranosyl-(1- > 2)-[4-hydroxycinnamoyl-(- > 6)-b-D-glucopyranosyl-(1- > 6)]-b-D-galactopyranoside] in cultivar 'Zihe' was significantly higher than the other two cultivars (Table S1). It is thus believed the two anthocyanins are responsible for the purple coloration in *R.pulchrum* [42, 43]. Leucodelphinidin 3-[Galactosyl -(1- > 4)-glucoside] concentration in dark colored petals was significantly higher than in light colored petals (Table S1). On the other hand, the amount of Cyanidin 3-O-(2 "-O-Galloyl-6"-O-alpha-rhamnopyranosyl-beta-galactopyranoside in light colored petals was significantly higher than in dark colored petals (Fig. 3b, Table S2). These results showed that Leucodelphinidin 3-[Galactosyl -(1- > 4)-glucoside] and Hesperetin 7-glucoside may contribute to the coloration of petals, while Malvidin 3-O-(6-O-(4-O-malonyl-alpha-rhamnopyranosyl)-beta-glucopyranoside)-5-O-beta-glucopyranoside,Cyanidin 3-O-[b-D-Xylopyranosyl-(1- > 2)-[4-hydroxycinnamoyl-(- > 6)-b-D-glucopyranosyl-(1- > 6)]-b-D-galactopyranoside], Medicarpin 3-O-(6'-malonylglucoside) and Hesperetin 7-neohesperidoside may contribute to the purple coloration of petals.

### Seven thousand seven hundred thirteen differentially expressed genes were found among three cultivars of *R.pulchrum*

**Fig. 4:**
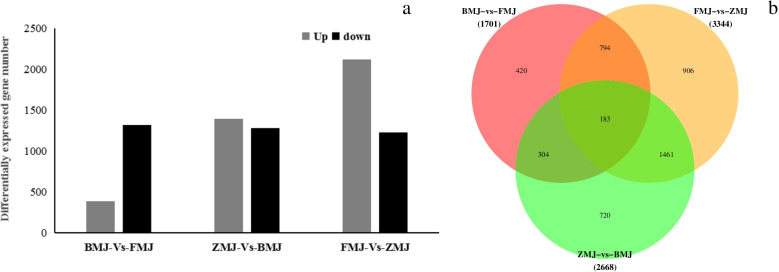
Differently expressed genes in *R.pulchrum* with different colors. Note: **a** Statistic of differently expressed gene in cultivars 'Baihe'(BMJ),'Fenhe'(FMJ) and 'Zihe'(ZMJ). **b** The Venn diagram of differently expressed gene between three *R.pulchrum* cultivars

To further investigate the molecular basis for flavonoid biosynthesis, transcriptome analysis was carried out to identify differentially expressed genes (DEGs) in petals of *R.pulchrum.* 61.84 Gb of clean data was produced from the petals of *R.pulchrum* (Table S3). The transcriptome data were then mapped to the genome of *Rhododendron simsii* and the mapping rate ranged from 84.21% to 85.27%. A total of 7713 DEGs were identified using the cut-off values of |log_2_ Fold change|≥ 1 and *p *Value < 0.05. In the comparison group between cultivars ‘Baihe’ (BMJ) and ‘Fenhe’ (FMJ), 381 DEGs were up-regulated while 1320 were down-regulated. Between the cultivars 'Zihe'(ZMJ) and 'Baihe'(BMJ) group, 1390 were up-regulated and 1278 were down-regulated. There were 2117 up-regulated genes and 1227 down-regulated genes in cultivar ‘Fenhe’ (FMJ) and ‘Zihe’ (ZMJ) group (Fig. 4).

### Comparative analysis of genes enriched by GO and KEGG in three comparison groups

**Fig.5:**
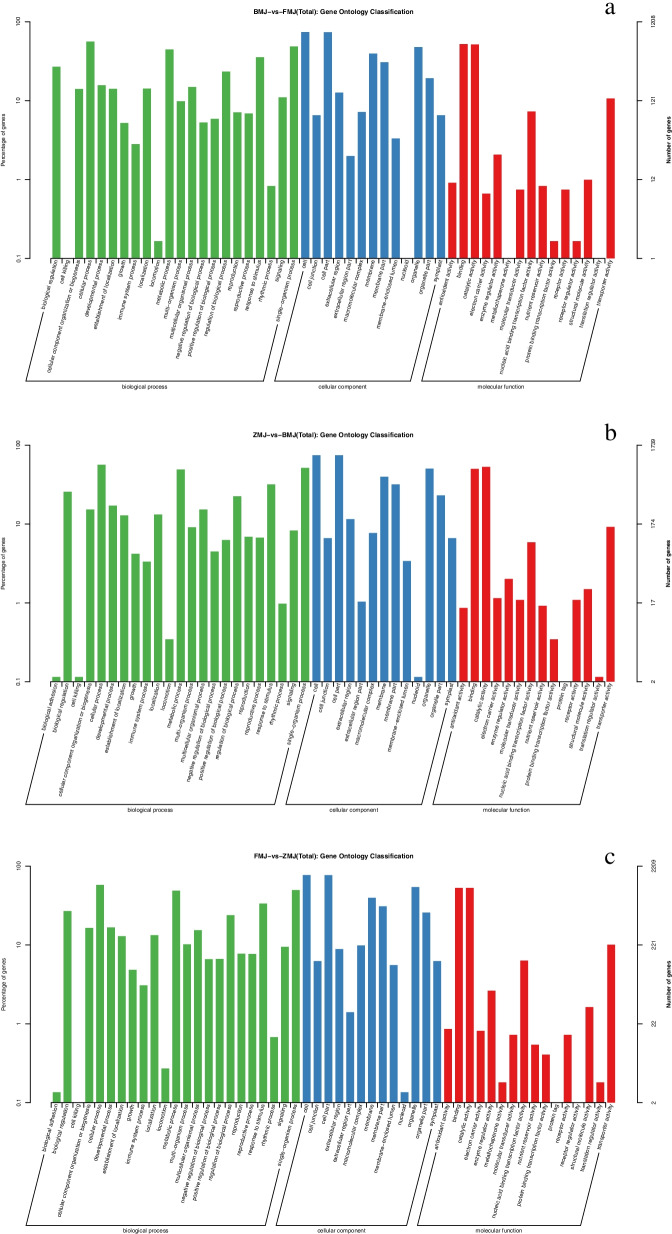
Transcript GO annotation classification statistics graph in three comparison groups. Note: BMJ, cultivar ‘Baihe’; FMJ, cultivar ‘Fenhe’; ZMJ, cultivar ‘Zihe’. **a** Transcript GO annotation classification between BMJ and FMJ; **b** Transcript GO annotation classification between ZMJ and BMJ; **c** Transcript GO annotation classification between FMJ and ZMJ

To understand their biological functions and gene interactions, 5156 of 7713 DEGs were annotated to GO database. 1208 and 1739 DEGs were categorized into 50 functional groups when cultivar ‘Baihe’ was compared with ‘Fenhe’ and ‘Zihe’, respectively. The number become to 2209 DEGs and 52 functional groups when comparing cultivar ‘Fenhe’ and ‘Zihe’. Three major GO categories were ‘Biological process’, ‘Cellular component’, and ‘Molecular function’. In detail, ‘Cellular process’, ‘Single-organism process’, and ‘Metabolic process’ were the most enriched terms in ‘Biological process’, ‘Cell’, ‘Cell part’, and ‘Organelle’ were the most enriched terms in ‘Cellular component’, while ‘Binding’, ‘Catalytic activity’, and ‘Transporter activity’ were the most enriched terms in ‘Molecular function’ (Fig. 5). 22 anthocyanin biosynthetic processes and 29 flavonoid biosynthetic processes related genes were identified in the ‘Biological process’, while two anthocyanin biosynthetic processes and one flavonoid biosynthetic process related genes were identified in the ‘Molecular function’.

**Fig. 6:**
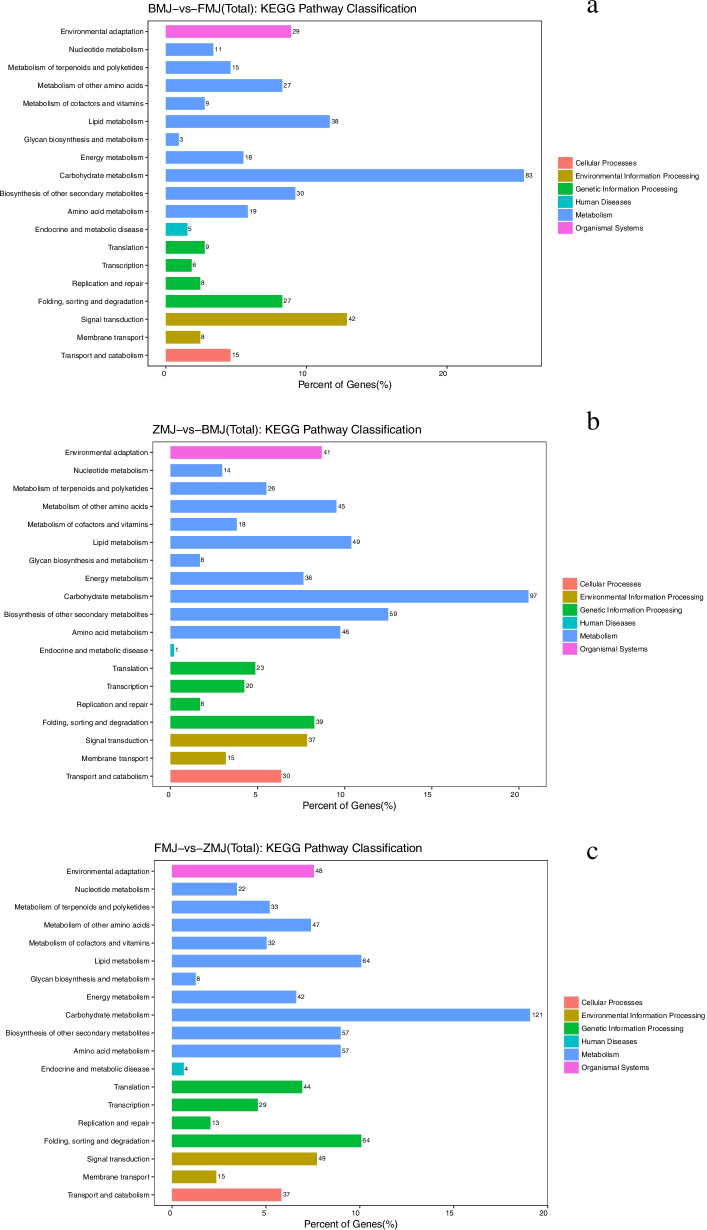
KEGG pathway classification statistics graph in three comparison groups. Note: BMJ, cultivar ‘Baihe’; FMJ, cultivar ‘Fenhe’; ZMJ, cultivar ‘Zihe’. **a** KEGG pathway classification statistics between BMJ and FMJ; **b** KEGG pathway classification statistics between ZMJ and BMJ; **c** KEGG pathway classification statistics between FMJ and ZMJ

Moreover, to identify the metabolic pathways involved in the flavonoids’ biosynthesis process, 1433 DEGs were annotated with the KEGG database. 326, 472, and 635 DEGs were enriched in 19 pathways belonging to 6 categories (Fig. 6). According to KEGG analysis, the top pathways were the metabolic pathways for carbohydrates, lipids, and other secondary metabolites. The classification showed that a large number of genes were enriched in the ‘Phenylpropanoid biosynthesis’(ko00940) and ‘Flavone and flavonol biosynthesis’(ko00944), which is important to the petal coloration (Table S4). Our study found DEGs enriched in the ‘Brassinosteroid biosynthesis pathway’(ko00905) in three comparison groups, and 22 DEGs were enriched in ‘Plant hormone signal transduction pathway’(ko04075) between cultivar ‘Baihe’ and ‘Fenhe’ group. Also, 9 DEGs were enriched in ‘Photosynthesis’(ko00195) between cultivars ‘Zihe’ and ‘Baihe’ group (Table S4). It was reported that plant hormones and light signals were closely related to the synthesis of anthocyanins [44, 45]. Our results indicated that plant hormones play an important role in white to pink coloration, while light signals play an active role in white to purple coloration in *R.pulchrum*.

### Specific transcription factor families enriched in the different color of *R.pulchrum*

**Fig. 7:**
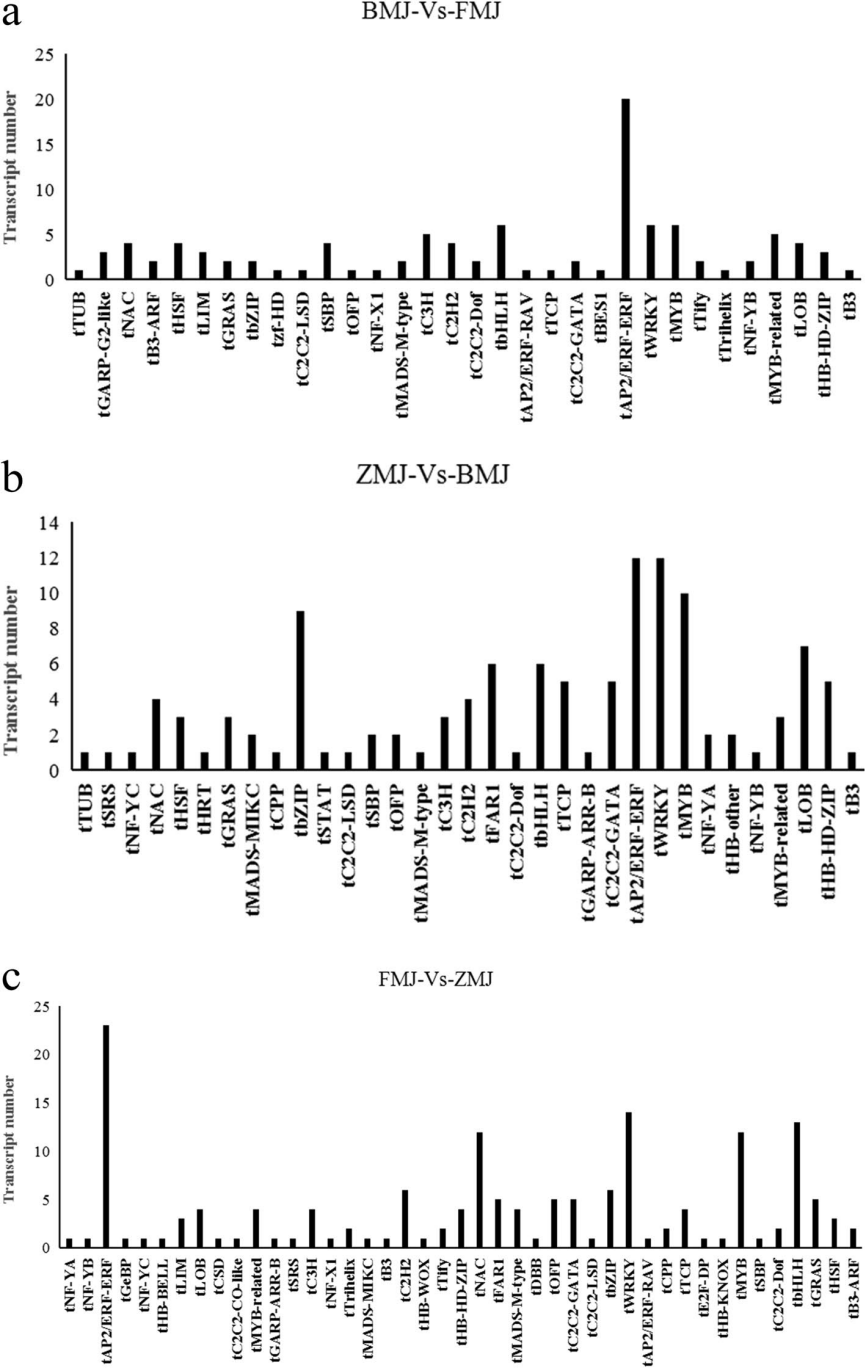
Distribution of transcription factor types in three comparison groups. Note: BMJ, cultivar ‘Baihe’; FMJ, cultivar ‘Fenhe’; ZMJ, cultivar ‘Zihe’. **a** Distribution of transcription factor types in BMJ and FMJ group; **b** Distribution of transcription factor types in ZMJ and BMJ group; **a** Distribution of transcription factor types in FMJ and ZMJ group

Transcription factors (TFs) play a critical role in the growth and development of plants by regulating gene expression. A total of 64 families of transcription factors were reported [46]. With transcriptome analysis, 32, 33 and 43 families of differentially expressed TFs were detected in the cultivars ‘Baihe’(BMJ) vs. ‘Fenhe’(FMJ), ‘Zihe’(ZMJ) vs. ‘Baihe’(BMJ), and ‘Fenhe’(FMJ) vs. ‘Zihe’ (ZMJ), respectively (Fig. 7). These TFs mainly belong to the *tAP2/ERF, MYB, WRKY, bHLH, MyB-related, NAC* and *bZIP* families (Fig. S3). In them, *WRKY* family was enriched in ‘Baihe’ and ‘Fenhe’, while *MADS-M* family was enriched in ‘Zihe’. ‘Fenhe’ had a high expression level of *tAP2/ERF* and *NAC* families. The *HB-HD-ZIP, FAR1,* and *OFP* families were highly expressed in ‘Zihe’. The results indicated that *tAP2/ERF* and *NAC* families were involved in the regulation of pink coloration, while *HB-HD-ZIP, FAR1 and OFP* families were involved in the purple coloration*.* The *HSF* family had a higher expression in ‘Baihe’. It was possible that *HSF* family inhibits color related gene expression. In addition, 16 genes were annotated as *MYB*-related genes, including *MYB5, MYB6, MYB15, MYB101, MYB73, MYB61, MYB308*, and other *MYB*-dominant proteins. 12 genes encoding *bHLH*. These TFs are associated with the flavonoid biosynthesis in the petals of *R.pulchrum*.

### Modeling of a new color-related flavonoid biosynthesis pathway in *R.pulchrum*

**Fig. 8:**
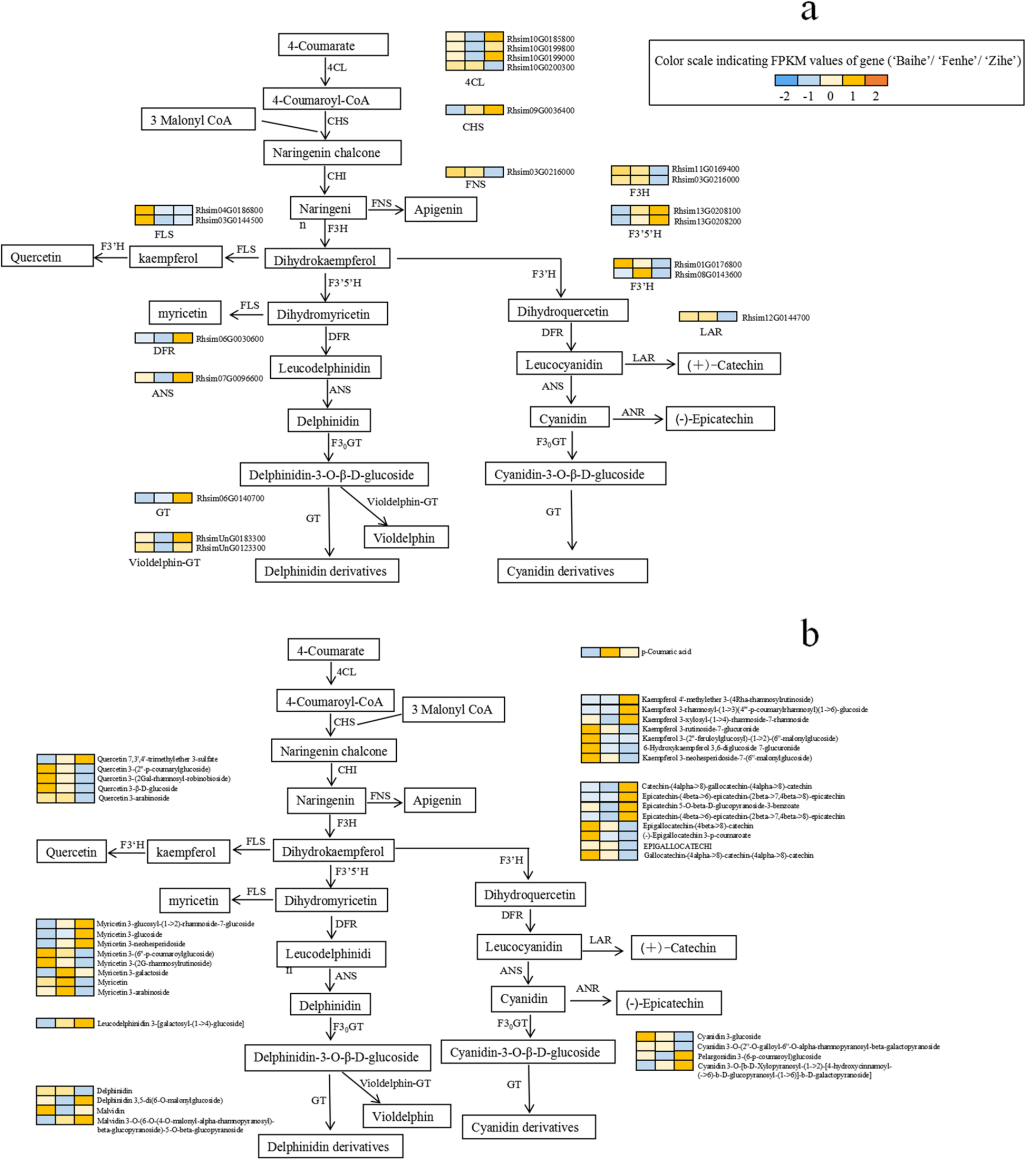
Biosynthesis pathway of flavonoid in three different color petals of *R.pulchrum* Sweet. Note: **a** Key structural genes and their expression level of involved in flavonoid biosynthesis pathway in *R.pulchrum* Sweet with different colors. **b** The expression levels of differential metabolites in flavonoid synthesis pathway

**Fig. 9:**
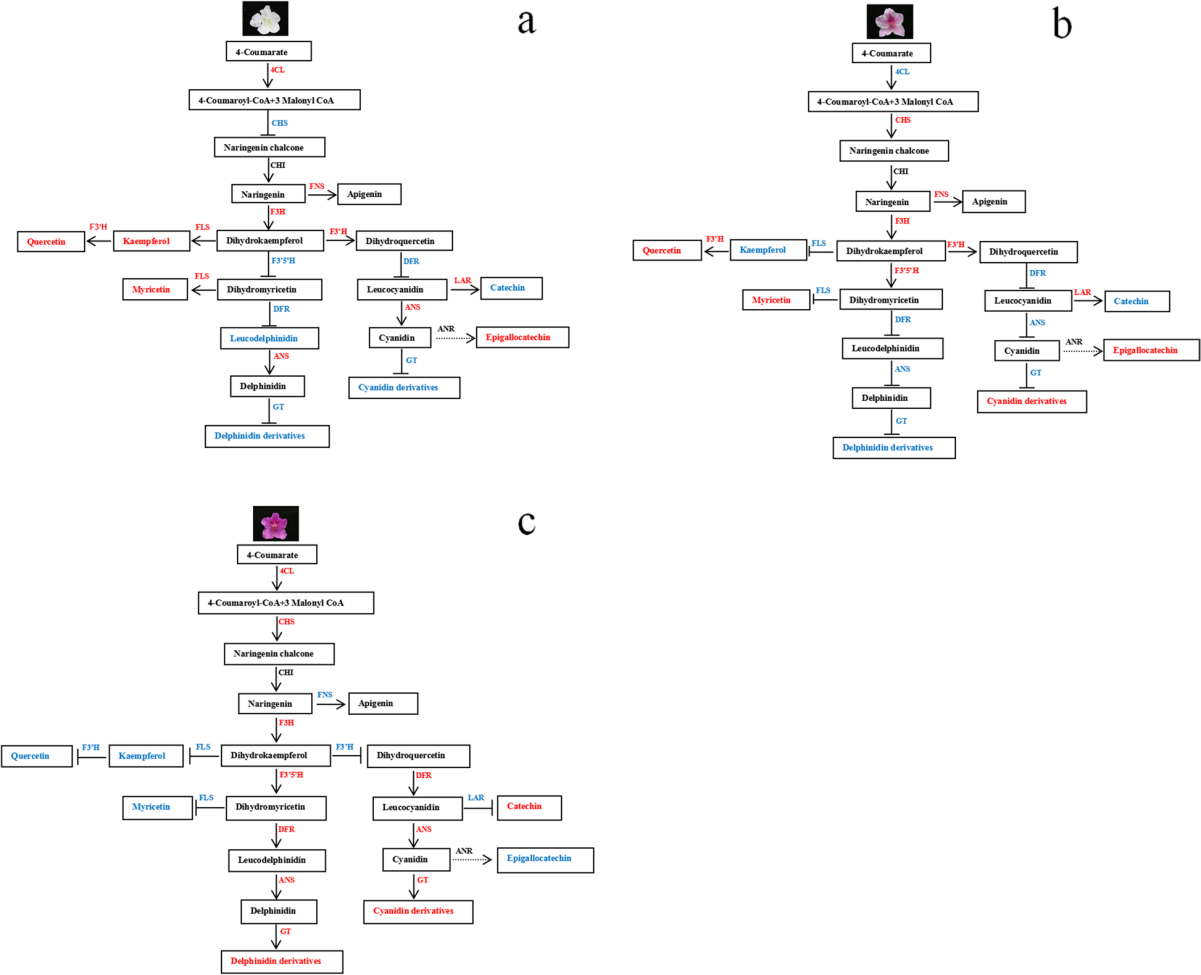
A model for the flavonoid biosynthesis pathway in three different color petals of *R.pulchrum* Sweet. Note: **a** cultivar ‘Baihe’. **b** cultivar ‘Fenhe’. **c** cultivar ‘Zihe’. The red color indicates genes and metabolites with high expression, and the blue color indicates genes and metabolites with low expression

Based on the expression patterns of key genes and metabolites, a model of the flavonoid biosynthesis pathway in *R.pulchrum* was constructed*.* In the modeling of cultivar ‘Baihe’, we speculated the inhibition of *CHS* gene led to a decrease in chalcone and naringin at the early stage (Fig. 8a). *FLS, F3′5'H*, and *F3'H* competed for the same substrate of dihydrokaempferol. Then, up-regulated of *FLS* and *F3'H* genes accompanied by down-regulated of *F3′5'H* gene drive the synthesis of kaempferol and quercetin derivatives in cultivar 'Baihe' (Fig. 8). Inhibition of *F3′5'H, DFR* and *GT* genes led to an inactivation of pigment accumulation, which resulted in the appearance of white petals (Fig. 9).

In the modeling of cultivar ‘Zihe’, *CHS* gene showed a higher expression level in 'Zihe' than in other two cultivars. Previous studies have reported that the high expression of *CHS* gene is responsible for purple anthocyanins in *Freesia hybrids* [47] and wheat [48]. *F3'H, F3′5'H, DFR, ANS, GT, and Violdelphin-GT* had higher expression levels while the *FLS, F3'H* and *LAR* genes with a lower expression in 'Zihe' compared with ‘Baihe’ and ‘Fenhe’ (Fig. 8a). The differential expression of *F3′5'H*, *FLS* and *F3'H* genes led to synthesize many delphinidins from dihydrokaempferol. It was found that delphinidin and malvidin derivatives were accumulated in 'Zihe', such as delphinidin 3,5-di(6-O-malonylglucoside) and malvidin3-O-(6-O-(4-O-malonyl-alpha-rhamnopyranosyl)-beta-glucopyranoside)-5-O-beta-glucopyranoside (Fig. 8b)*.* The low expression of *F3'H* gene and the high expression of *DFR, ANS* and *GT* genes leading to accumulation of epicatechins and cyanidins. In the meantime, cyanidin could be converted to cyanidin and pelargonidin glycosides catalyzed by *GT.* The metabolome analysis found pelargonidin 3-(6-p-coumaroyl) glucoside and cyanidin3-O-[b-D-Xylopyranosyl-(1- > 2)-[4-hydroxycinnamoyl-(- > 6)-b-D-glucopyranosyl-(1- > 6)]-b-D-galactopyranoside] were enriched in cultivar 'Zihe'. These DEGs are significantly associated with the production of reddish purple pigmentation in 'Zihe' (Fig. 9).

In the modeling of ‘Fenhe’, coumaric acid expression was higher, but the low expression of the *4CL* gene restrained the accumulation of 4-Coumaroyl-CoA at the early stage of flavonoid synthesis pathway (Fig. 8b). The expression of *FLS* gene is inhibited, meanwhile the *F3'H* and *F3′5'H* genes were highly expressed, which led to overaccumulation of dihydromyricetin and dihydroquercetin (Fig. 8a). Because of the inhibition of the *DFR, ANS*, and *GT* genes, the delphinidin and cyanidin synthesis pathways are shut down leading to the formation of light purplish pink petals in cultivar 'Fenhe'. (Fig. 9).

To validate the key genes related to flavonoid biosynthesis, nine genes were randomly selected for further verification with RT-PCR (Table S6). It was found that six genes (*4CL, CHS, ANS, F3 ′5' H, GT, Viodelphin-GT*) were up-regulated in cultivar 'Zihe', three genes (*F3H, FLS, LAR*) were up-regulated in cultivars ‘Baihe’ and ‘Fenhe’. These results were consistent with what we discovered from RNA-seq (Fig. S4).

The integrated transcriptome and metabolome analysis was performed to gain a deeper understanding of the color-specific genes. 10 DEMs and 6 DEGs, 36 DEMs and 22 DEGs, and 37 DEMs and 23 DEGs enriched in flavonoid pathway were selected from the comparison groups of 'Baihe' vs. 'Fenhe', 'Zihe' vs. 'Baihe', and 'Fenhe' vs. 'Zihe', respectively. A Pearson correlation analysis of gene expression and metabolite response intensity was performed [49, 50]. *CHS, F3′5'H, ANS*, and *Viodelphin-GT* were highly expressed, and *LAR* had a low expression in purple petals, and the metabolites associated with these genes were also enriched in the petals (Table S5). Our integrative analysis suggested that differential expression of *CHS, FLS, F3' H, F3 ′5 'H*, *DFR*, *GT, FAOMT* in the flavonoid biosynthesis caused different colors between white and purple. In addition, *F3' H* and *F3 ′5 'H* up-regulated, *4CL, DFR, ANS* and *GT* down-regulated are significantly associated with pink coloration.

### Color-specific modules and hub genes were identified by using WGCNA

**Fig. 10:**
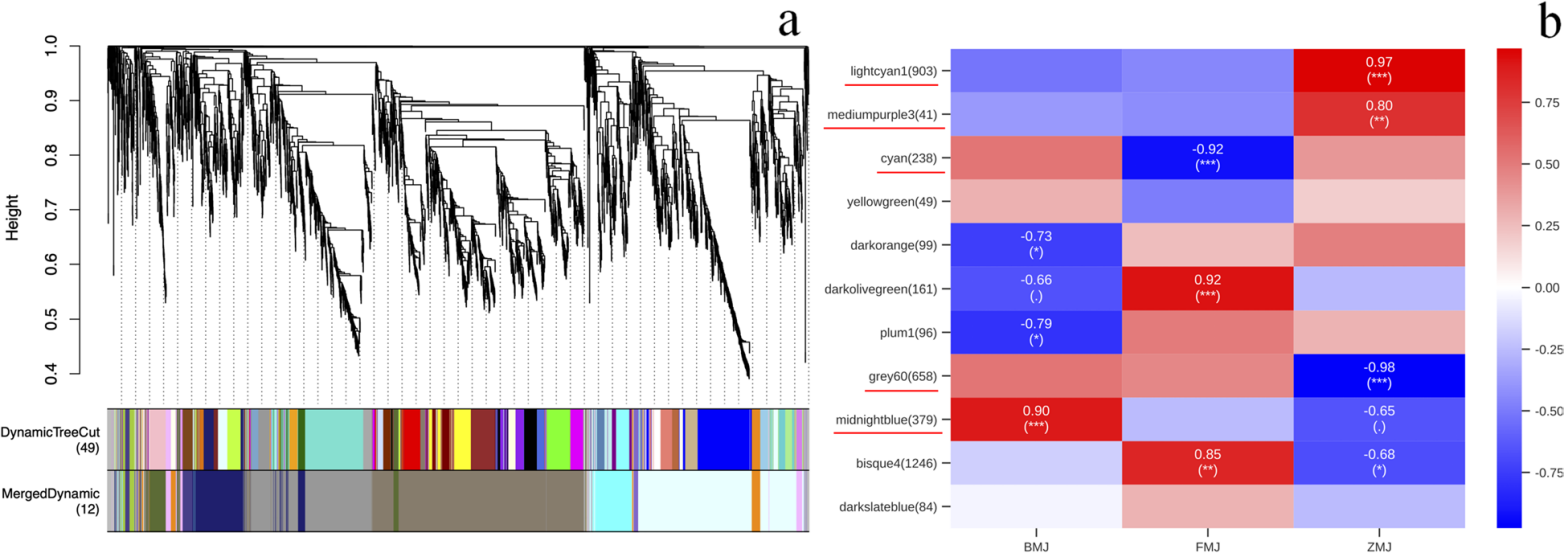
WGCNA of genes in three different color petals of *R.pulchrum* Sweet. **a** Hierarchical clustering tree (cluster dendrogram) results showed 12 expression modules, labeled with different colors. **b** Module–sample association. Each row corresponds to a module, labeled with a color as in (**a**). The number of genes in each module is indicated on the left

**Fig. 11:**
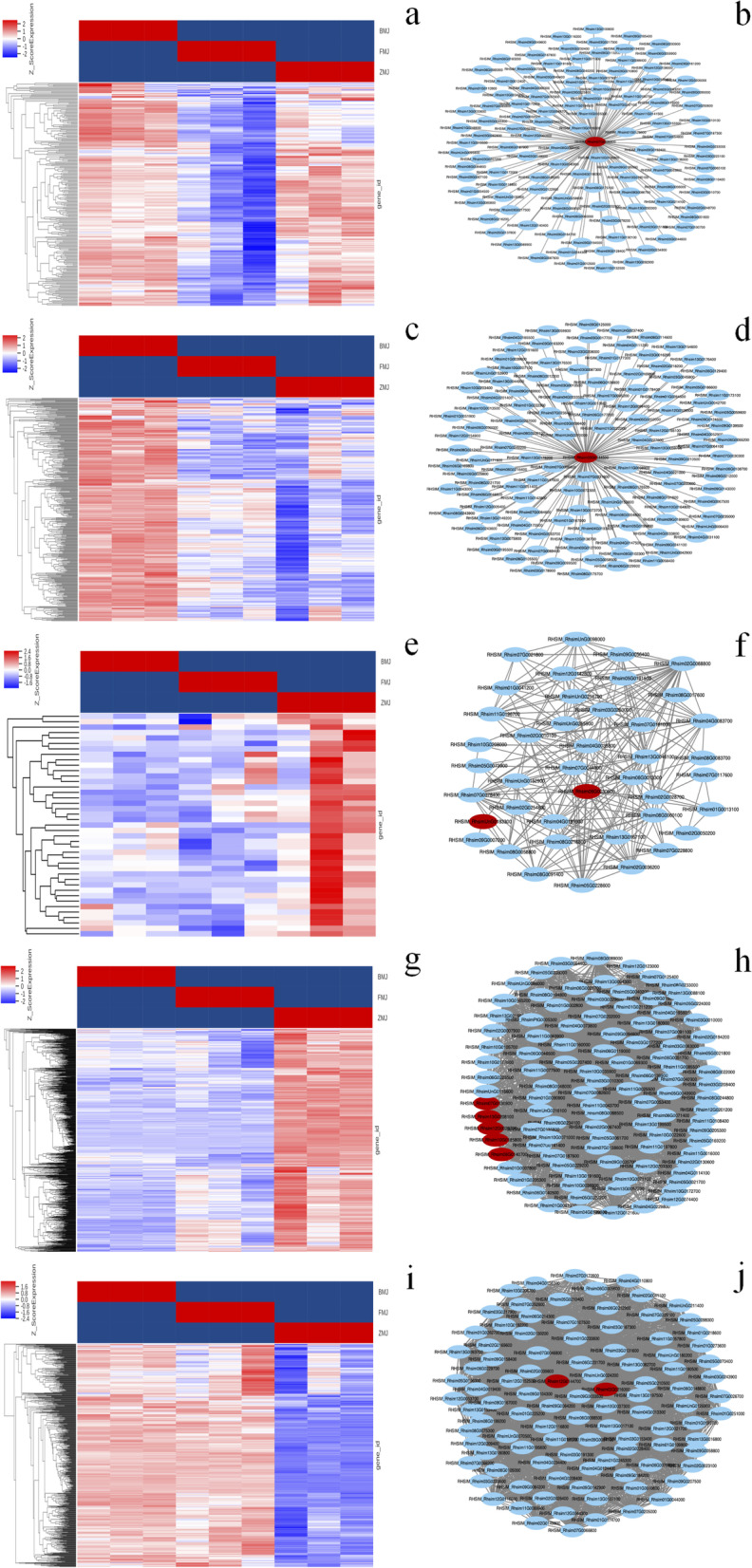
Coexpression network analysis in three different color petals of *R.pulchrum* Sweet. (**a**), (**c**), (**e**), (**g**), and (**i**) Heatmaps showing genes in cyan, midnightblue, mediumpurple3, lightcyan1, and grey60 modules that were significantly over-represented in BMJ, FMJ, and ZMJ, respectively. (**b**), (**d**), (**f**), (**h**), and (**j**) The correlation networks in the module corresponding to (**a**), (**c**), (**e**), (**g**), and (**i**), respectively. Candidate hub genes are shown in red

To further validated the flavonoid biosynthesis related key genes, a WGCNA (Weighted Correlation Network Analysis) was performed [51]. After filtering out the genes with a low expression (FPKM < 0.05), 4066 genes were obtained for the WGCNA. 12 distinct modules were identified (labeled with different colors) shown in the dendrogram in Fig. 10a. Heat map of the 12 modules are shown in Fig. 10b. The lightcyan1, mediumpurple3 and grey60 modules with 903, 41 and 658 genes, were highly associated with the cultivars 'Zihe' (*r* = 0.97, 0.80 and -0.98). *GT*, *FAOMT*, *F3′5’H*, *4CL*, *IFR* genes are enriched in the lightcyan1 module, and *DFR* and *Viodelphin-GT* genes were enriched in mediumpurple3 module, while the grey60 module contained *LAR* and *FNS* genes. The cyan module with 238 genes was highly associated with cultivars 'Fenhe' (r = -0.92), in which *ANS* was enriched. The midnightblue module with 379 genes was highly associated with cultivars 'Baihe' (*r *= 0.90), which contained *FLS* gene (Fig. 11). The WGCNA results suggested that *ANS, FLS, LAR, FNS, GT, 4CL, F3′5’H, IFR, DFR*, *FAOMT* and *Viodelphin-GT* genes are associated with the coloration of petals in *R.pulchrum*, in which the genes of *ANS, FLS, LAR, GT, 4CL, F3′5’H, DFR*, *FAOMT* and *Viodelphin-GT* are found in the integrated analysis of transcriptome and metabolome. The results indicated these hubs genes are significantly important for the coloration of *R.pulchrum.*

## Discussion

Multi-omics analysis can be used to study the molecular mechanisms of plant coloration [36]. Multiple metabolites are mainly accumulated in flavonoid biosynthesis, flavonoid and flavonol biosynthesis, and anthocyanin biosynthesis pathways in *R.pulchrum*. We provided a model illustrating the color related key genes and metabolites and their regulatory mechanism in *R. pulcherum.*

### Floral color and key genes in flavonoid biosynthesis of *R.pulchrum*

At the early stage of flavonoid biosynthesis, *4CL* and *CHS* genes showed differential expression levels among three cultivars, but not happened with *CHI* gene. 4-Coumarate CoA Ligase (*4CL*) participates in the metabolism of a variety of secondary compounds in plants, such as lignin, coumarin, flavonoids, anthocyanins, and phenols [52–54]. We had identified four genes that encode 4-Coumarate CoA Ligase, three of them (*Rhsim10G0185800, Rhsim10G0199800, and Rhsim10G0199000*) are highly expressed in cultivar ‘Zihe’, and the other one (*Rhsim10G0200300*) is highly expressed in cultivar ‘Fenhe’. The high expression of these genes contributed to the accumulation of more precursor compounds in cultivar 'Zihe'. Chalcone synthase (*CHS*), as a key initiating enzyme in the flavonoid synthesis pathway, exists in a multi-gene family in most species [55]. *CHS* expression was significantly lower in cultivar 'Baihe' compared with 'Fenhe' and 'Zihe', which resulted in the inhibition of flavonoid biosynthesis in 'Baihe' at the early stage. *CHI* gene did not show differential expressions in different color petals. As Takos et al. discovered there was no difference in *CHI* expression between the peels of red and non-red apple varieties, but when *CHI* was completely absent, anthocyanin synthesis was limited [56]. Therefore, they believed *CHI* was not a key enzyme for the catalytic synthesis of chalcone naringin, but rather acted as rate-limiting enzyme in the flavonoid pathway.

At the late stage of flavonoid biosynthesis, *FLS, F3H, F3 'H, DFR, LAR, F3 ′5' H, ANS, GT*, and *violdelphin-GT* genes showed differential expression in three petals. Flavonol synthase (*FLS*), a key enzyme in the biosynthesis of flavonols, catalyzes the production of kaempferol and myricetin from dihydrokaempferol and dihydromyricetin respectively. A deletion of *FLS* or an alteration of gene expression can affect the coloration of plant organs [57, 58]. As a result of high FLS expression in cultivar 'Baihe', high levels of kaempferol and quercetin derivatives were found. *FLS* play a more active role in catalyzing the dihydrokaempferol than *F3'H* [59], resulting in the accumulation of kaempferol and quercetin derivatives in 'Baihe'. It was reported that *FLS* and *DFR* compete for substrates to synthesize flavonols, and an upregulation of *FLS* and a downregulation of *DFR* may result in a reduction of anthocyanin accumulation [60], this is in line with our findings. There are *MYB* transcription factor binding sites and light signal response sites in most *FLS* gene promoters, indicating that *FLS* gene transcription is regulated by *MYB* transcription factors and light signals [61, 62]. In this study, the DEGs are enriched in the 'light signaling pathway' in 'Fenhe' and 'Zihe' group (Table S4). However, whether the *FLS* gene is regulated by light signal and *MYB* transcription factors in *R.pulchrum* needs further study. The flavonoid 3 'hydroxylase (*F3'H*) is a critical enzyme in producing Purplish Red [63]. As well as catalyzing kaempferol to form quercetin, it also catalyzes dihydrokaempferol to form dihydroquercetin. There are two genes that encode *F3 'H* (*Rhsim01G0176800* and *Rhsim08G0143600*). *Rhsim01G0176800* was highly expressed in cultivar 'Baihe', whereas *Rhsim08G0143600* was highly expressed in cultivar 'Fenhe'. We speculate that these two genes may have distinct regulatory pathways, *Rhsim01G0176800* catalyzes kaempferol to produce quercetin, while *Rhsim08G0143600* catalyzes dihydrokaempferol to produce dihydroquercetin, further validations are required. *F3 ′5' H* is a key gene associated with the synthesis of blue color. In our study, *F3 ′5' H* (*Rhsim13G0208100*, *Rhsim13G0208200*) are highly expressed in cultivar 'Zihe', leading to the synthesis of dihydromyricetin, a substrate of delphinidin and malvidin glycosides. Both *LAR* and *ANS* play an important role in the synthesis of proanthocyanins and anthocyanins, which are downstream genes in the flavonoid biosynthesis pathway. It is known that leucocyanidin is the substrate of *LAR* and *ANS* genes, which could catalyze leucocyanidin into catechins and cyanins, respectively [45]. *LAR* gene expression is affected by temperature, light, and hormones [64], and *ANS* gene expression is closely linked to plant organ coloration [65, 66]. In our finding, *LAR* was highly expressed in cultivars ‘Fenhe’ and ‘Baihe’, resulting in the synthesis of more Catechin derivatives using leucocyanidin. *ANR* and *GT* were competing for substrate cyanins. The enzyme A*NR* catalyzes the production of epicatechin from cyanins, which is also affected by light, temperature, and hormones [64]. By catalyzing the glycosylation of anthocyanin, flavonoid 3-O-glucosyltransferase (*GT*) is an important enzyme in the last stage of anthocyanin synthesis [67]. Interestingly, there was no difference in *ANR* expression among the three cultivars, but cultivar 'Zihe' exhibited a higher concentration of epicatechin derivatives, suggesting that the use of cyanidin as a substrate to synthesize epicatechin derivatives resulted in accumulation of less red pigmentation.

### Floral color and *MYBs, AP2/ERFs* transcription factors in *R.pulchrum*

*MYB* is one of the largest transcription factor families in plants. It mainly regulates the flavonoid biosynthesis via two pathways. Firstly, *MYBs* activate the expression of flavonoid synthesis genes by interacting with *bHLH*s and *WD40*s to form the *MBW* complex [68]. *FaMYB5* may act as negative regulators of PA/ anthocyanin biosynthesis in strawberry [69]. *MYB5* (*Rhsim01G0174700*) was highly expressed in cultivars 'Baihe' and 'Fenhe', suggesting that it may negatively regulate anthocyanin synthesis. Secondly, *MYBs* can regulate the expression of structural genes independently to influence flavonoid synthesis. As reported by Luo et al., *FTMYB15* was transferred from *Tartary Buckwheat* to *Arabidopsis thaliana,* the pigment accumulation was found in the leaves and seed coat of the transgenic plant [70]. *MYB15* (*Rhsim04G0061700*) was discovered with downregulation in cultivar ‘Zihe’. It is suggested that it may inhibit purple coloration in *R. pulcherum*. According to previous studies, *MYB6* regulates flavonol biosynthesis through activating the promoters of *F3H* and *FLS* [71]. Based on our findings, *MYB6* (*Rhsim07G0002700*) was highly expressed in 'Fenhe', and rutin content in 'Fenhe' was significantly higher than in 'Zihe'. It is indicated *MYB6* was also involved in the pink coloration of petals through the regulation of flavonol synthesis. The *MYB101* participated in pollen tube reception and leaf development [72–74]. According to our results, *MYB101* (*Rhsim06G0163100*) was highly expressed in cultivar ‘Fenhe’, suggesting that *MYB101* may be responsible for regulating flavonoids in *R.pulchrum* with pink petals.

UV-B can be used as a signal to regulate plant development, such as regulating photomorphogenesis and promoting the accumulation of flavonoids and anthocyanins [75–77]. *MYB73* is an important transcription factor associated with plant hormones [78]. UVR8 interacts with *MYB73/MYB77* (MYB DOMAIN PROTEIN 73/77) in a UV-B-dependent manner, it can affect plant growth and development through light and auxin signal [79]. When *MYB73* (*Rhsim05G0088000*) was highly expressed in cultivar 'Zihe', *MYB73* may contribute to the accumulation of flavonoids and anthocyanins in *R.pulchrum* by regulating the photomorphogenesis process. *MYB61 (Rhsim11G0106400)* was highly expressed in dark flowers, suggesting that it may regulate the coloration in *R.pulchrum*.

The *AP2/ERF* family is one of the largest transcription factors families in plants and regulate primary and secondary metabolism [80–82]. It has been shown that *AP2/ERF* can regulate the biosynthesis of anthocyanins in plants. *MdMYB1* is a positive regulator of anthocyanin biosynthesis, *and MdERF38* interacts with *MdMYB1* to promote the binding of *MdMYB1* to its target gene in the drought-induced conditions [83]. *DREB* subfamily, which belongs to *AP2/ERF* family, was only found to be highly expressed in cultivar 'Fenhe'. But further research is needed to confirm whether *DREB* can regulate flavonoid biosynthesis in pink petals by affecting the interactions of TFs under certain induction conditions. *Rhsim07G0115900* and *Rhsim03G0161100,* which encode *ERF5,* were discovered in this study. *Rhsim07G0115900* was lowly expressed in cultivar ‘Zihe’ when compared with ‘Fenhe’ and ‘Baihe’, while *Rhsim03G0161100* was highly expressed in cultivar ‘Baihe’ when compared with ‘Fenhe’. It is suggested that *ERF5* might be involved in regulation of flavonoid biosynthesis in *R. pulcherum*.

### The *FNS* and *IFR* genes were identified in *R.pulchrum*

Flavone synthases had two different subtypes, Flavone synthase I (*FNSI*) and Flavone synthase II (*FNSII*). *FNSI* is a nonheme iron-dependent a-ketoglutarate dioxygenase, which is found in the cytosol [84]. As a member of the P450 family, *FNSII* is found in the inner membrane of cells. Both enzymes can convert naringin to apigenin, and they are not simultaneously in the same species. Researcher has reported that *Medicago sativa* and *soybean* contain only *FNSII* [71, 85]. Metabolome analysis revealed that the apigenin derivatives had a lower expression in cultivar 'Zihe' than in 'Baihe' and 'Fenhe’. It was found that *Rhsim03G0216000*, which encodes *FNS*, was lowly expressed in cultivar 'Zihe'. Sequence alignment results revealed that *Rhsim03G0216000* had a great similarity (74%) to *FNSI*(*AT5G24531.1*). Based on these findings, it is likely that *FNS* is in the form of *FNSI* in *R.pulchrum*.

Isoflavone is a type of phytoestrogens, which have only been found in some species of Papilionoideae [86, 87]. The isoflavone reductase (*IFR*) catalyzes the conversion of isoflavones to Medicarpin, which is the first step in isoflavone decomposition [88, 89]. The *IFR* gene (*Rhsim07G0136900*) was highly expressed in cultivar 'Zihe', meanwhile metabolite of isoflavones (Medicarpin3-O-(6'-malonylglucoside)) highly enriched in cultivar 'Zihe'. Therefore, it is necessary to investigate whether the isoflavone derivatives cause the purple coloring of *R.pulchrum*. Medicalarpin is a natural phytoalexin of the pterocarpan subfamily. It has been found in many traditional Chinese medicines, such as *Pueraria Lobata* [90]and *Hedysari Radix* [91]. The studies have reported that it has various biologically benefits, including stimulation of bone regeneration, induction of apoptosis, and induction of lipolysis [92]. Interesting, many medicarpin derivatives have been found in cultivar 'Zihe' for the first time, suggesting that this cultivar is valuable for medicine research. It has been observed that a few purple spots are sometimes on the white petals of *R.pulchrum*, we discovered delphinidin and malvidin were accumulated in the petals. The blockage of glycosylation related genes in the flavonoid synthesis pathway may explain why white flowers accumulated some delphinidin and malvidin.

## Conclusion

The transcriptome and metabolome analysis were used to identify genes, transcription factors, and metabolites associated with the coloration of *R.pulchrum*. For known color-related transcription factors, the *DREB* family were found to play a critical role in the formation of the pink color. Additionally, our results have shown that *FNS* and *IFR* were highly expressed in the flavonoid biosynthesis pathway, and that *FNS* exists in the form of *FNSI* in *R.pulchrum*. The *IFR* gene and its related metabolites of medicarpin derivatives were highly expressed in cultivar 'Zihe', indicating that *IFR* involved in the purple coloration of *R.pulchrum*. *CHS, FLS, F3' H, F3 ′5 'H, DFR*, *GT* and *FAOMT* genes were correlated with white and purple coloration, whereas F3' H and *F3 ′5 'H* up-regulated, *4CL*, *DFR, ANS* and *GT* down-regulated were associated with pink coloration. We provided a model illustrating the color related key genes and metabolic and their regulatory mechanism of *R. pulcherum.* The study provided some basis for breeding new cultivars with different colors of petals.

## Methods

### Plant materials and treatment

**Fig. 12:**
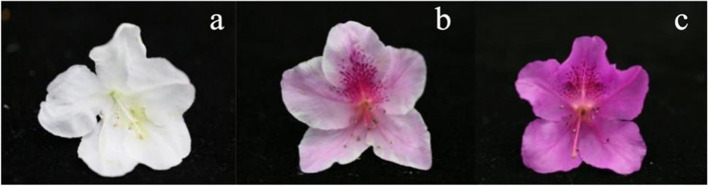
Three different colors of *Rhododendron pulchrum* Sweet. Note: **a**. ‘Baihe’; **b**.‘Fenhe’; **c**.‘Zihe’

The white-flowered *R.pulchrum* Sweet ('Baihe'), pink-flowered *R.pulchrum* Sweet ('Fenhe') and purple-flowered *R.pulchrum* Sweet ('Zihe') were grown at the *Rhododendron* resource center in the Shanghai Botanical Garden (31°15′ N, 121°45′ E), Shanghai, China (Fig. 12). After collecting the petals from ‘Baihe’,’Fenhe’ and’Zihe’, all materials were frozen in liquid nitrogen and stored at − 80 °C for RNA and metabolite extraction. In the transcriptome analysis of ‘Baihe’, ‘Fenhe’ and ‘Zihe’, for each color, we gathered three samples as biological replicates, then each sample randomly selected three blooms petals from the same plant and pooled into one for RNA-Seq. For metabolism analysis, we had made five biological replicates from five individual plant for each color.

### Sample preparation and metabolite extraction

Each color of petal was collected from five individual plants. 1 mL of methanol: water (7:3, v:v) was added to 80 mg samples. 2-chloro-l-phenylalanine (20μL, 0.3 mg / mL) was added as an internal standard. All samples were precooled at -20 °C for 2 min, pulverized with grinder at 60 HZ for 2 min, then ultrasonicated for 30 min and placed at -20 °C for 20 min. The samples were centrifuged for 10 min (13,000 rpm, 4 °C). The supernatants (150μL) from each tube were collected with crystal syringes, filtered through 0.22 μm microfilters, and injected into LC vials. Until the liquid chromatography–mass spectrometry (LC–MS) analysis was performed, the vials were stored at -80 °C. A pooled QC sample was prepared by mixing aliquots of all the samples. For the metabolic profiling, an ACQUITY UHPLC system (Waters Corporation, Milford, USA) in conjunction with an AB SCIEX Triple TOF 5600 System (AB SCIEX, Framingham, MA) was used. ACQUITY UPLC BEH C18 columns (1.7 μm, 2.1 × 100 mm) were used in both positive and negative mode. The binary gradient elution system consisted of (A) water (containing 0.1% formic acid, v/v) and (B) acetonitrile (containing 0.1% formic acid, v/v). The gradient program consisted of 0 min, 95% A; 2 min, 80% A; 4 min, 75% A; 9 min, 40% A; 17 min, 0% A; 19 min, 0% A; 19.1 min, 95% A; and 20.1 min, 95% A. The flow rate was 0.4 mL/min and the injection volume were 5μL. The temperature of the column was maintained at 45 °C. During the LC–MS analysis, samples were maintained in the instrument chamber at 4 °C.

### Metabolite identification and quantification

The metabolome data was analyzed using Progenesis QI software (Nonlinear Dynamics, Newcastle, United Kingdom). Simca software package (version 14.0, Umetrics, Umeå, Sweden) was used to analyze the combined positive and negative data. Human Metabolome (http://www.hmdb.ca/), METLIN (http://metlin.scripps.edu) and LIPID MAPS (http://www.lipidmaps.org/) databases were used for metabolite identification. An orthogonal partial least squares discriminant analysis (OPLS-DA) was conducted to identify potential biomarker variables. Significantly different metabolites between groups were determined by variable importance in projection (VIP) ≥ 1 and an absolute Log_2_FC (foldchange) ≥ 1. A hierarchical clustering analysis was conducted with R (http://www.r-project.org/).

### RNA-seq and annotation

For each sample, 1 g fully opened stage petals were used for extracting the RNA using the mirVana miRNA Isolation Kit (Ambion). RNA purity and quantification were assessed using a NanoDrop 2000 spectrophotometer (Thermo Scientific, USA). RNA integrity was evaluated using an Agilent 2100 Bioanalyzer (Agilent Technologies, Santa Clara, CA, USA). Following the manufacturer's instructions, libraries were constructed using TruSeq Stranded mRNA LTSample Prep Kit (Illumina, San Diego, CA, USA). Afterwards, these libraries were sequenced on the Illumina sequencing platform (Illumina HiSeq X Ten) and 150 bp paired-end reads were obtained. Raw reads were first processed using Trimmomatic [93], and low-quality reads and adaptors were filtered out. HISAT2 [94] was used to map the remaining reads to the reference genome. The differential expression analysis was carried out using the DESeq R package [95]. According to the DESeq analysis, genes with adjusted P-values of < 0.05 were significantly differentially expressed. A hierarchical cluster analysis of DEGs was performed to analyze gene expression patterns. GO enrichment and KEGG [96] pathway enrichment analysis of DEGs were performed with R based on the hypergeometric tests. The annotation of gene functions was carried out using the following databases: Kyoto Encyclopedia of Genes and Genomes (KEGG), eukaryotic Clusters of Orthologous Groups (KOG), NCBI non-redundant (NR), Swiss PROT sequence protein, Gene Ontology (GO) and the homologous protein family (Pfam). Three biological replicates of each sample were performed.

### RT-qPCR verification

A total of nine structural genes related to flavonoid biosynthesis were selected for validation by quantitative real-time PCR (RT-qPCR). Total RNA was extracted with Total RNA Kit (Ambion).The RNA was treated with DNase I(Invitrogen) to remove trace genomic DNA, then reverse-transcribed in a GeneAmp® PCR System 9700 (Applied Biosystems, USA).The specific primers were designed in the laboratory and synthesized by TsingKe Biotech (Supplementary Table S6). RT-qPCR was performed using a LightCycler® 480 II Real-time PCR with Instrument (Roche, Swiss).The PCR amplification was 94℃ for 30 s, followed by 45 cycles of 94℃ for 5 s, 60℃ for 30 s. The expression levels of mRNAs were normalized to GAPDH and were calculated using the 2^−ΔΔCt^ method [97]. The primers of RT-qPCR are showed in Table S6.

### Metabolites-transcripts correlation and weighted gene coexpression network analysis

The Pearson correlation algorithm was used to calculate the correlation between genes and metabolites. DEGs and DEMs have been mapped simultaneously to the KEGG Pathway database to obtain information on their pathways. The correlation analysis was performed by R 3.5.1 using Pearson correlation analysis. Coexpression networks was performed with WGCNA analysis [51].

## Supplementary Information

**Additional file 1: Fig. S1.** Volcano plot of differential metabolits in R.pulchrum Sweet.(a) volcano plot of differential metabolits between cultivars ‘Baihe’ and ‘Fenhe’.(b)volcano plot of differential metabolits between cultivars ‘Zihe’ and ‘Baihe’.(c)volcano plot of differential metabolits between cultivars ‘Fenhe’ and ‘Zihe’.

**Additional file 2: Fig. S2.** Hierarchical cluster analysis of differential flavonoid metabolites in R.pulchrum Sweet. Note: White, cultivar ‘Baihe’; Pink, cultivar ‘Fenhe’; Purple, cultivar ‘Zihe’. (a)Analysis of flavonoid metabolites between cultivars ‘Baihe’ and ‘Fenhe’;(b) Analysis of flavonoid metabolites between cultivars ‘Zihe’ and ‘Baihe’; (c) Analysis of flavonoid metabolites between cultivars ‘Fenhe’ and ‘Zihe’.

**Additional file 3: Fig. S3.** Transcription factor distribution between three R.pulchrum Sweet cultivars. Note: BMJ, cultivar ‘Baihe’; FMJ, cultivar ‘Fenhe’; ZMJ, cultivar ‘Zihe’. (a) Transcription factor distribution between cultivars ‘Baihe’ and ‘Fenhe’; (b) Transcription factor distribution between cultivars ‘Zihe’ and ‘Baihe’; (c) Transcription factor distribution between cultivars ‘Fenhe’ and ‘Zihe’.

**Additional file 4: Fig. S4.** Transcript accumulation measurements of colour-related genes involved in the flavonoid metabolic process. Note: BMJ, cultivar ‘Baihe’; FMJ, cultivar ‘Fenhe’; ZMJ, cultiv.

**Additional file 5: Table S1.** Flavonoid metabolites between three comparison groups of R.pulchrum Sweet.

**Additional file 6: Table S2.** The anthocyanin derivatives between three comparison groups of R.pulchrum Sweet.

**Additional file 7: Table S3.** Transcriptome sequencing results from three R.pulchrum Sweet cultivars.

**Additional file 8: Table S4.** Enrichment of TOP20 pathways in R.pulchrum Sweet based on KEGG analysis.

**Additional file 9: Table S5.** Association analysis of DEGs and DEMs in R.pulchrum Sweet.

**Additional file 10: Table S6.** The primers used for qRT-PCR analysis.

## Data Availability

All relevant supporting data sets are included in the article and its supplemental files.

## Abbreviations

PAL: Phenylalanine ammonia-lyase
4CL: 4-Coumarate:coenzyme A ligase
CHS: Chalcone synthase
CHI: Chalcone isomerase
F3H: Flavanone 3-hydroxylase
FLS: Flavonol synthase
F3′H: Flavanone 3′-hydroxylase
F3′5’H: Flavonoid 3′, 5′- hydroxylase
DFR: Dihydroflavonol 4-reductase
ANS: Anthocyanidin synthase
LAR: Leucoanthocyanidin reductase
ANR: Anthocyanidin reductase
GT: Glucosyltransferase
Violdelphin-GT: Viodelphin-glucosyltransferases
F3_0_GT: Flavonol-3-O-glucosyl transferase
COG: Clusters of orthologous groups of proteins database
DEG: Differentially expressed gene
DEM: Differentially expressed metabolite
FPKM: Fragments per kilobase of exon model per million mapped reads
GO: Gene Ontology
KEGG: Kyoto Encyclopedia of Genes and Genomes
MYB: V-myb avian myeloblastosis viral oncogene homolog
bHLH: Basic helix-loop-helix
RNA-Seq: RNA sequencing
RT-qPCR: Real-time quantitative polymerase chain reaction
NGS: Next generation sequencing
HCA: Hierarchical cluster analysis
LC–MS: Liquid chromatography–mass spectrometry
WGCNA: Weighted correlation network analysis
