# Supplementary material for: Integrative analysis of transcriptome and metabolome reveals flavonoid biosynthesis regulation in Rhododendron pulchrum petals
Source: BMC Plant Biol. 2022 Aug 16;22:401. doi: 10.1186/s12870-022-03762-y (PMC9380304; doi:10.1186/s12870-022-03762-y)
Supplement: Supplementary file 1 — Additional file 1: Fig. S1. Volcano plot of differential metabolits in R.pulchrum Sweet.(a) volcano plot of differential metabolits between cultivars ‘Baihe’ and ‘Fenhe’.(b)volcano plot of differential metabolits between cultivars ‘Zihe’ and ‘Baihe’.(c)volcano plot of differential metabolits between cultivars ‘Fenhe’ and ‘Zihe’. [file 12870_2022_3762_MOESM1_ESM.pdf]

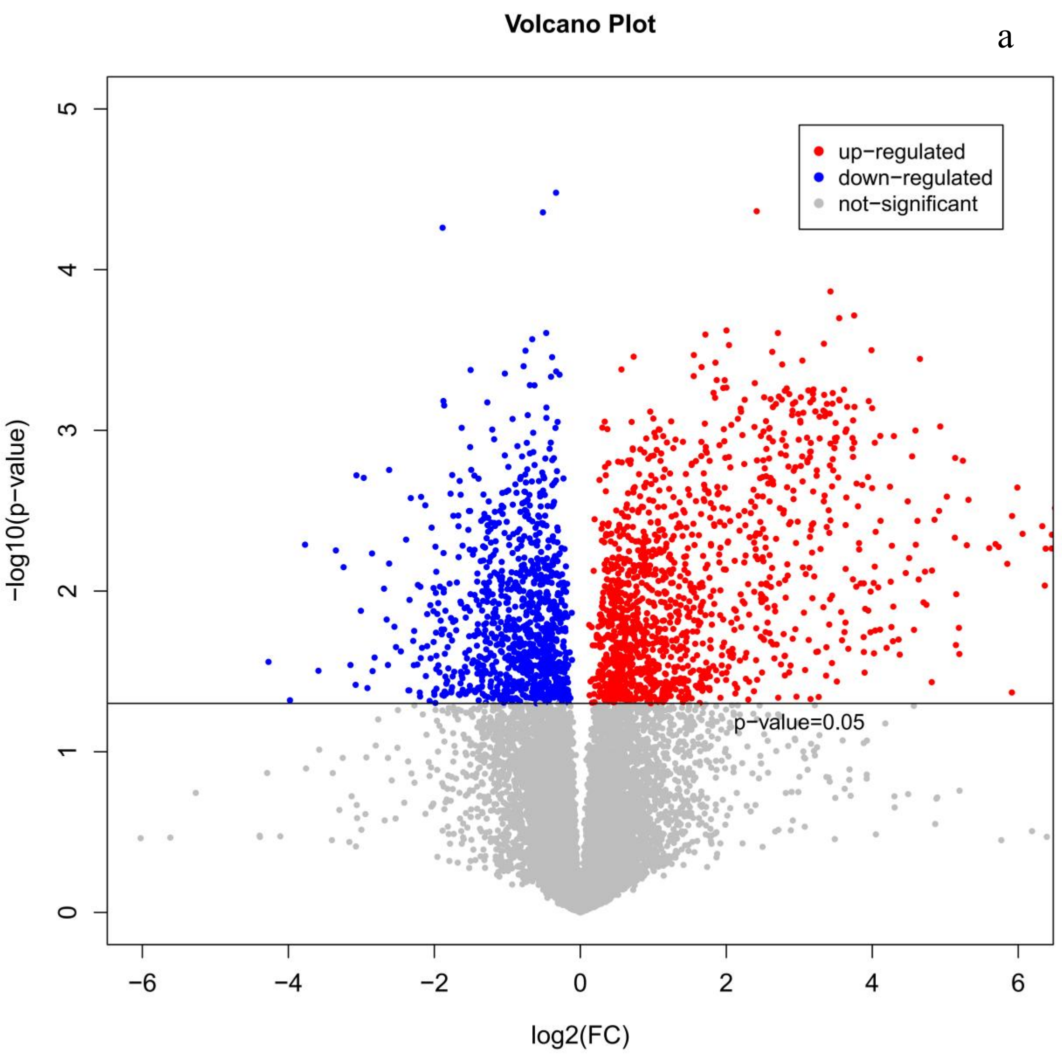

Volcano Plot
a
up−regulated
down−regulated
not−significant
p−value=0.05
−log10(p−value)
log2(FC)
0
1
2
3
4
5
−6
−4
−2
0
2
4
6

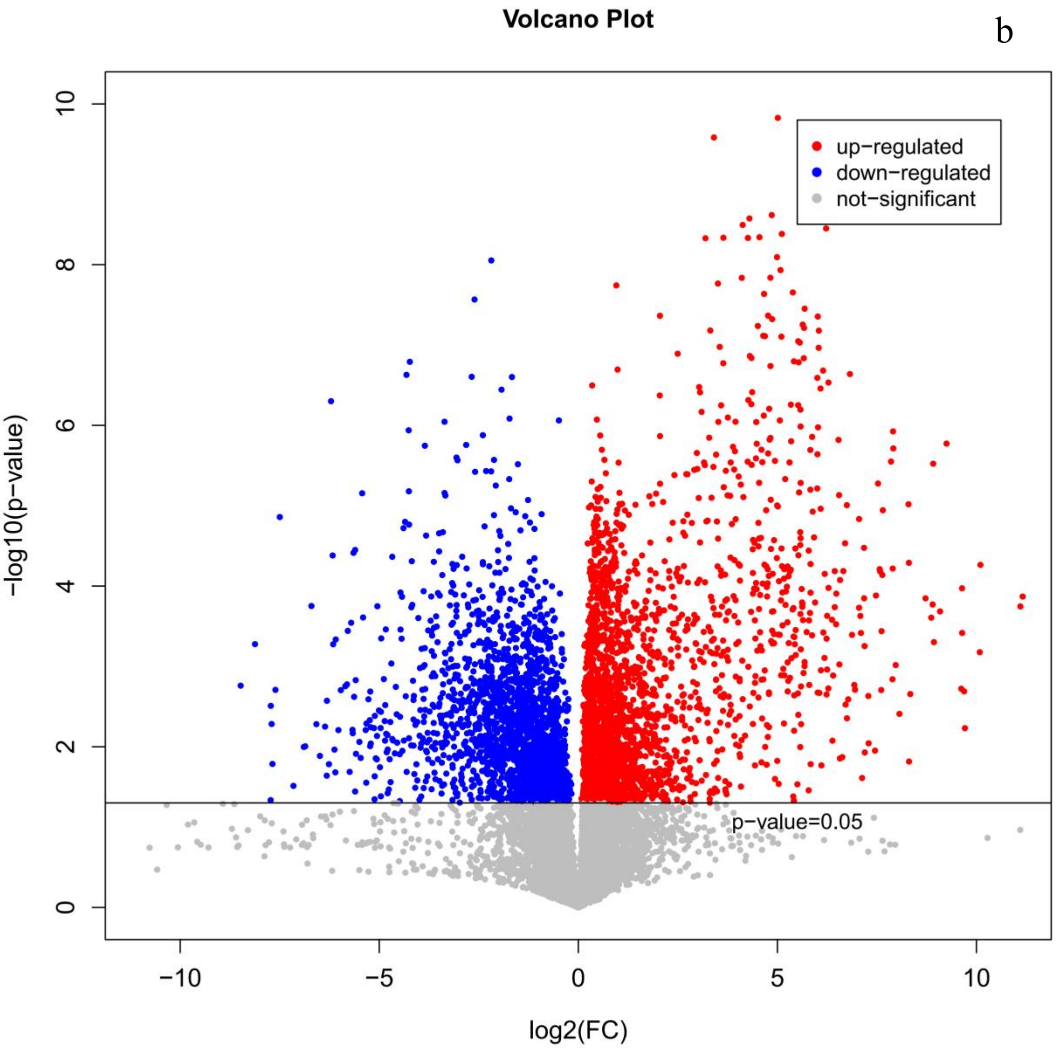
Volcano Plot
b
up−regulated
down−regulated
not−significant
p−value=0.05
−log10(p−value)
log2(FC)
0
2
4
6
8
10
−10
−5
0
5
10

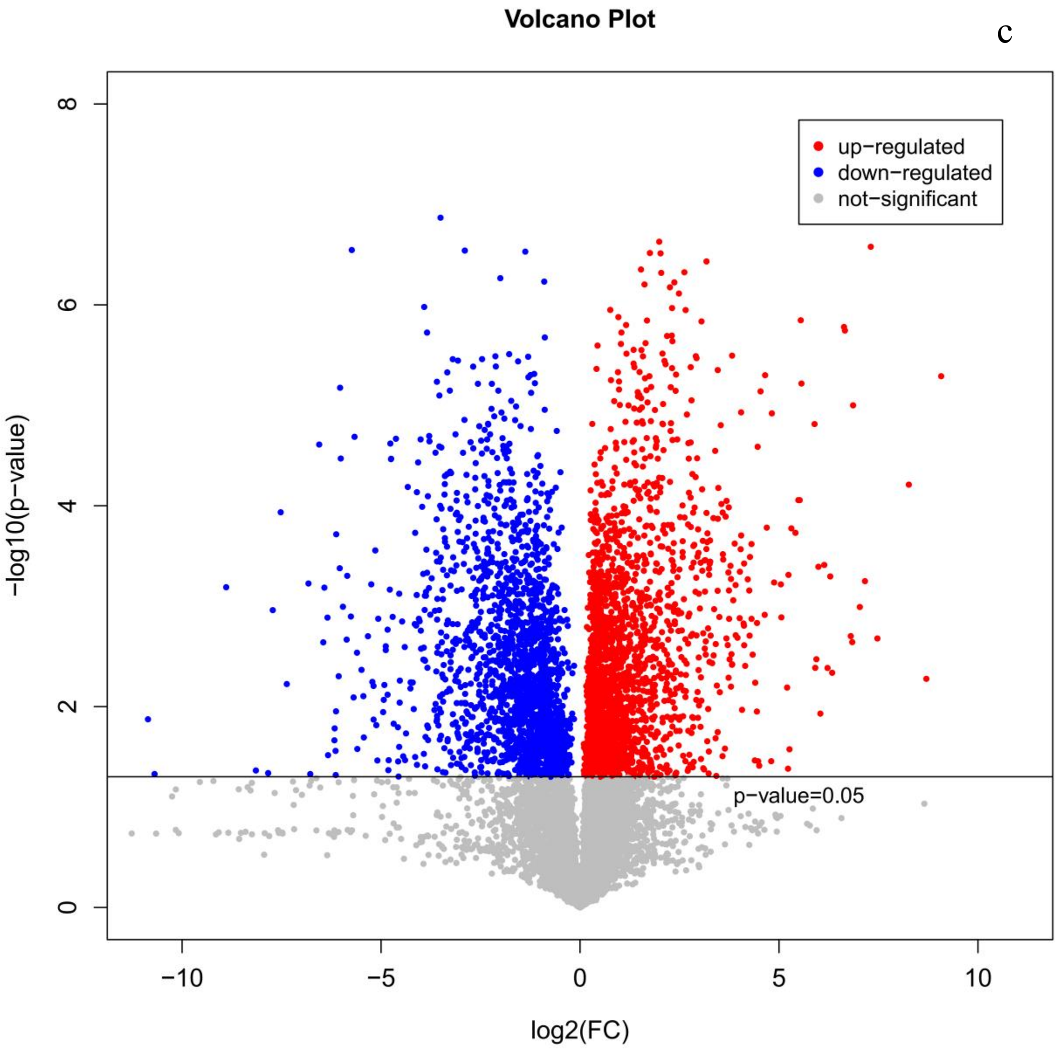

Fig.S1 Volcano plot of differential metabolits in *R.pulchrum* Sweet.(a) volcano plot of differential metabolits between cultivars ‘Baihe’ and ‘Fenhe’.(b)volcano plot of differential metabolits between cultivars ‘Zihe’ and ‘Baihe’.(c)volcano plot of differential metabolits between cultivars ‘Fenhe’ and ‘Zihe’.
