# Supplementary material for: Integrative analysis of transcriptome and metabolome reveals flavonoid biosynthesis regulation in Rhododendron pulchrum petals
Source: BMC Plant Biol. 2022 Aug 16;22:401. doi: 10.1186/s12870-022-03762-y (PMC9380304; doi:10.1186/s12870-022-03762-y)
Supplement: Supplementary file 2 — Additional file 2: Fig. S2. Hierarchical cluster analysis of differential flavonoid metabolites in R.pulchrum Sweet. Note: White, cultivar ‘Baihe’; Pink, cultivar ‘Fenhe’; Purple, cultivar ‘Zihe’. (a)Analysis of flavonoid metabolites between cultivars ‘Baihe’ and ‘Fenhe’;(b) Analysis of flavonoid metabolites between cultivars ‘Zihe’ and ‘Baihe’; (c) Analysis of flavonoid metabolites between cultivars ‘Fenhe’ and ‘Zihe’. [file 12870_2022_3762_MOESM2_ESM.pdf]

a

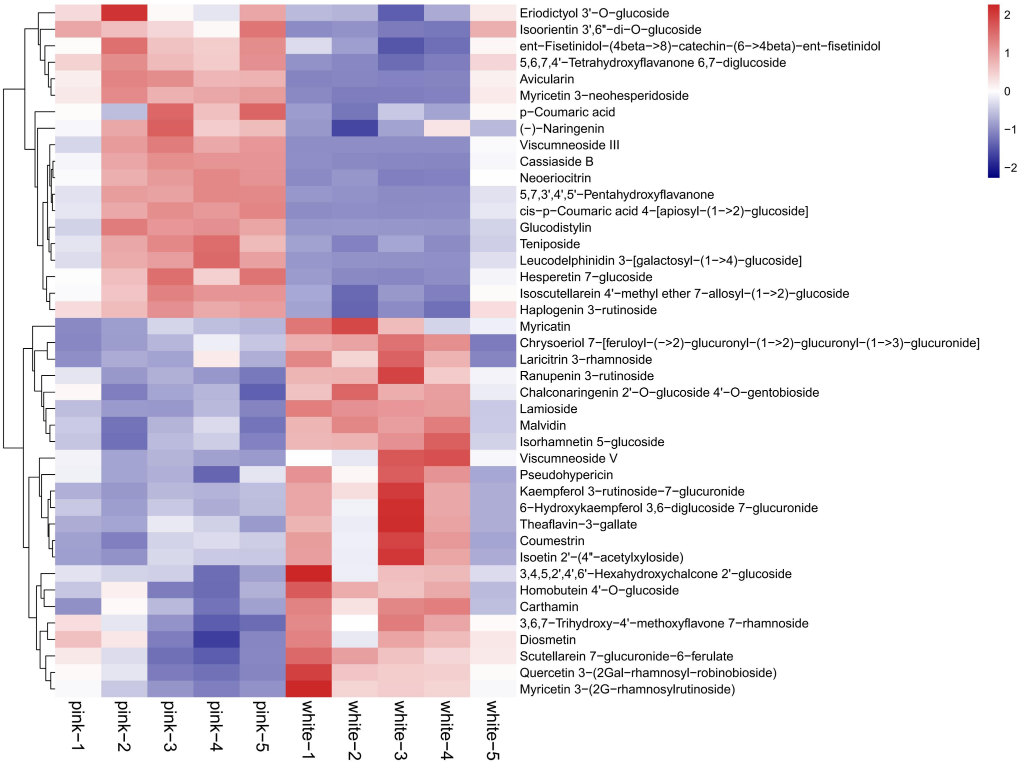
Eriodictyol 3'−O−glucoside
Isoorientin 3',6"−di−O−glucoside
ent−Fisetinidol−(4beta−>8)−catechin−(6−>4beta)−ent−fisetinidol
5,6,7,4'−Tetrahydroxyflavanone 6,7−diglucoside
Avicularin
Myricetin 3−neohesperidoside
p−Coumaric acid
(−)−Naringenin
Viscumneoside III
Cassiaside B
Neoeriocitrin
5,7,3',4',5'−Pentahydroxyflavanone
cis−p−Coumaric acid 4−[apiosyl−(1−>2)−glucoside]
Glucodistylin
Teniposide
Leucodelphinidin 3−[galactosyl−(1−>4)−glucoside]
Hesperetin 7−glucoside
Isoscutellarein 4'−methyl ether 7−allosyl−(1−>2)−glucoside
Haplogenin 3−rutinoside
Myricatin
Chrysoeriol 7−[feruloyl−(−>2)−glucuronyl−(1−>2)−glucuronyl−(1−>3)−glucuronide]
Laricitrin 3−rhamnoside
Ranupenin 3−rutinoside
Chalconaringenin 2'−O−glucoside 4'−O−gentobioside
Lamioside
Malvidin
Isorhamnetin 5−glucoside
Viscumneoside V
Pseudohypericin
Kaempferol 3−rutinoside−7−glucuronide
6−Hydroxykaempferol 3,6−diglucoside 7−glucuronide
Theaflavin−3−gallate
Coumestrin
Isoetin 2'−(4"−acetylxyloside)
3,4,5,2',4',6'−Hexahydroxychalcone 2'−glucoside
Homobutein 4'−O−glucoside
Carthamin
3,6,7−Trihydroxy−4'−methoxyflavone 7−rhamnoside
Diosmetin
Scutellarein 7−glucuronide−6−ferulate
Quercetin 3−(2Gal−rhamnosyl−robinobioside)
Myricetin 3−(2G−rhamnosylrutinoside)
pink−1
pink−2
pink−3
pink−4
pink−5
white−1
white−2
white−3
white−4
white−5
2
1
0
−1
−2

b

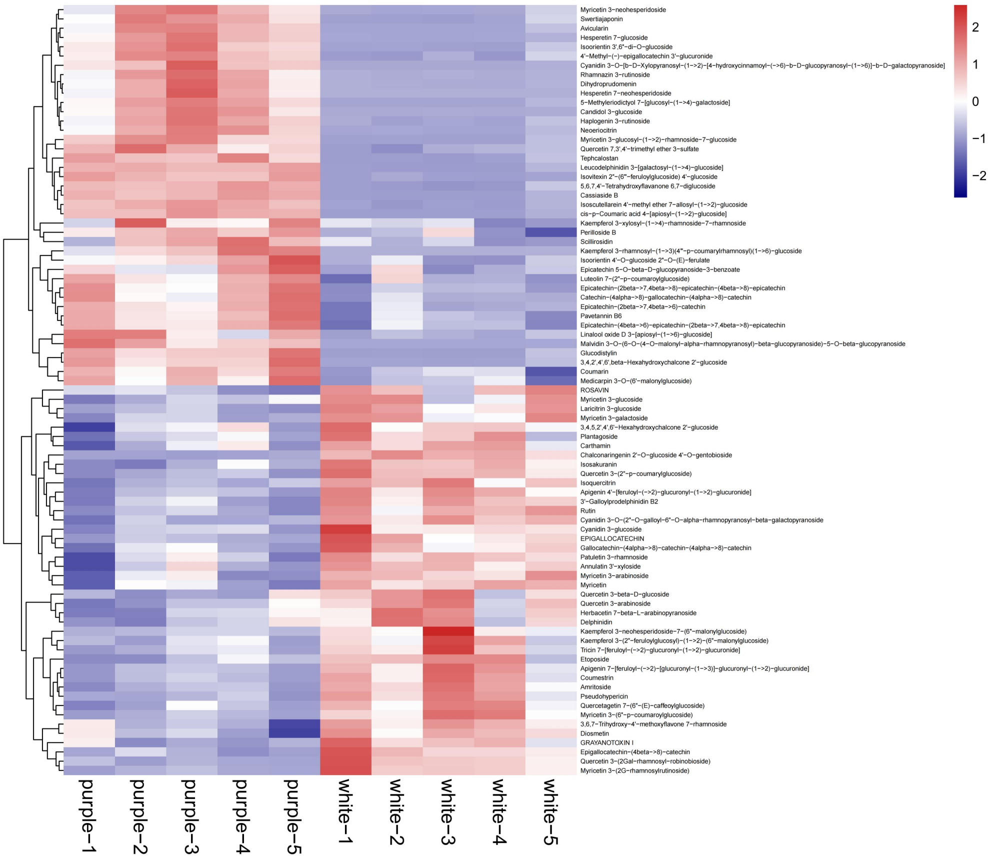
Myricetin 3−neohesperidoside
Swertiajaponin
Avicularin
Hesperetin 7−glucoside
Isoorientin 3',6"−di−O−glucoside
4'−Methyl−(−)−epigallocatechin 3'−glucuronide
Cyanidin 3−O−[b−D−Xylopyranosyl−(1−>2)−[4−hydroxycinnamoyl−(−>6)−b−D−glucopyranosyl−(1−>6)]−b−D−galactopyranoside]
Rhamnazin 3−rutinoside
Dihydroprudomenin
Hesperetin 7−neohesperidoside
5−Methyleriodictyol 7−[glucosyl−(1−>4)−galactoside]
Candidol 3−glucoside
Haplogenin 3−rutinoside
Neoeriocitrin
Myricetin 3−glucosyl−(1−>2)−rhamnoside−7−glucoside
Quercetin 7,3',4'−trimethyl ether 3−sulfate
Tephcalostan
Leucodelphinidin 3−[galactosyl−(1−>4)−glucoside]
Isovitexin 2"−(6'''−feruloylglucoside) 4'−glucoside
5,6,7,4'−Tetrahydroxyflavanone 6,7−diglucoside
Cassiaside B
Isoscutellarein 4'−methyl ether 7−allosyl−(1−>2)−glucoside
cis−p−Coumaric acid 4−[apiosyl−(1−>2)−glucoside]
Kaempferol 3−xylosyl−(1−>4)−rhamnoside−7−rhamnoside
Perilloside B
Scillirosidin
Kaempferol 3−rhamnosyl−(1−>3)(4'''−p−coumarylrhamnosyl)(1−>6)−glucoside
Isoorientin 4'−O−glucoside 2"−O−(E)−ferulate
Epicatechin 5−O−beta−D−glucopyranoside−3−benzoate
Luteolin 7−(2"−p−coumaroylglucoside)
Epicatechin−(2beta−>7,4beta−>8)−epicatechin−(4beta−>8)−epicatechin
Catechin−(4alpha−>8)−gallocatechin−(4alpha−>8)−catechin
Epicatechin−(2beta−>7,4beta−>6)−catechin
Pavetannin B6
Epicatechin−(4beta−>6)−epicatechin−(2beta−>7,4beta−>8)−epicatechin
Linalool oxide D 3−[apiosyl−(1−>6)−glucoside]
Malvidin 3−O−(6−O−(4−O−malonyl−alpha−rhamnopyranosyl)−beta−glucopyranoside)−5−O−beta−glucopyranoside
Glucodistylin
3,4,2',4',6',beta−Hexahydroxychalcone 2'−glucoside
Coumarin
Medicarpin 3−O−(6'−malonylglucoside)
ROSAVIN
Myricetin 3−glucoside
Laricitrin 3−glucoside
Myricetin 3−galactoside
3,4,5,2',4',6'−Hexahydroxychalcone 2'−glucoside
Plantagoside
Carthamin
Chalconaringenin 2'−O−glucoside 4'−O−gentobioside
Isosakuranin
Quercetin 3−(2"−p−coumarylglucoside)
Isoquercitrin
Apigenin 4'−[feruloyl−(−>2)−glucuronyl−(1−>2)−glucuronide]
3'−Galloylprodelphinidin B2
Rutin
Cyanidin 3−O−(2"−O−galloyl−6"−O−alpha−rhamnopyranosyl−beta−galactopyranoside
Cyanidin 3−glucoside
EPIGALLOCATECHIN
Gallocatechin−(4alpha−>8)−catechin−(4alpha−>8)−catechin
Patuletin 3−rhamnoside
Annulatin 3'−xyloside
Myricetin 3−arabinoside
Myricetin
Quercetin 3−beta−D−glucoside
Quercetin 3−arabinoside
Herbacetin 7−beta−L−arabinopyranoside
Delphinidin
Kaempferol 3−neohesperidoside−7−(6"−malonylglucoside)
Kaempferol 3−(2"−feruloylglucosyl)−(1−>2)−(6"−malonylglucoside)
Tricin 7−[feruloyl−(−>2)−glucuronyl−(1−>2)−glucuronide]
Etoposide
Apigenin 7−[feruloyl−(−>2)−[glucuronyl−(1−>3)]−glucuronyl−(1−>2)−glucuronide]
Coumestrin
Amritoside
Pseudohypericin
Quercetagetin 7−(6"−(E)−caffeoylglucoside)
Myricetin 3−(6"−p−coumaroylglucoside)
3,6,7−Trihydroxy−4'−methoxyflavone 7−rhamnoside
Diosmetin
GRAYANOTOXIN I
Epigallocatechin−(4beta−>8)−catechin
Quercetin 3−(2Gal−rhamnosyl−robinobioside)
Myricetin 3−(2G−rhamnosylrutinoside)
purple−1
purple−2
purple−3
purple−4
purple−5
white−1
white−2
white−3
white−4
white−5
2
1
0
−1
−2

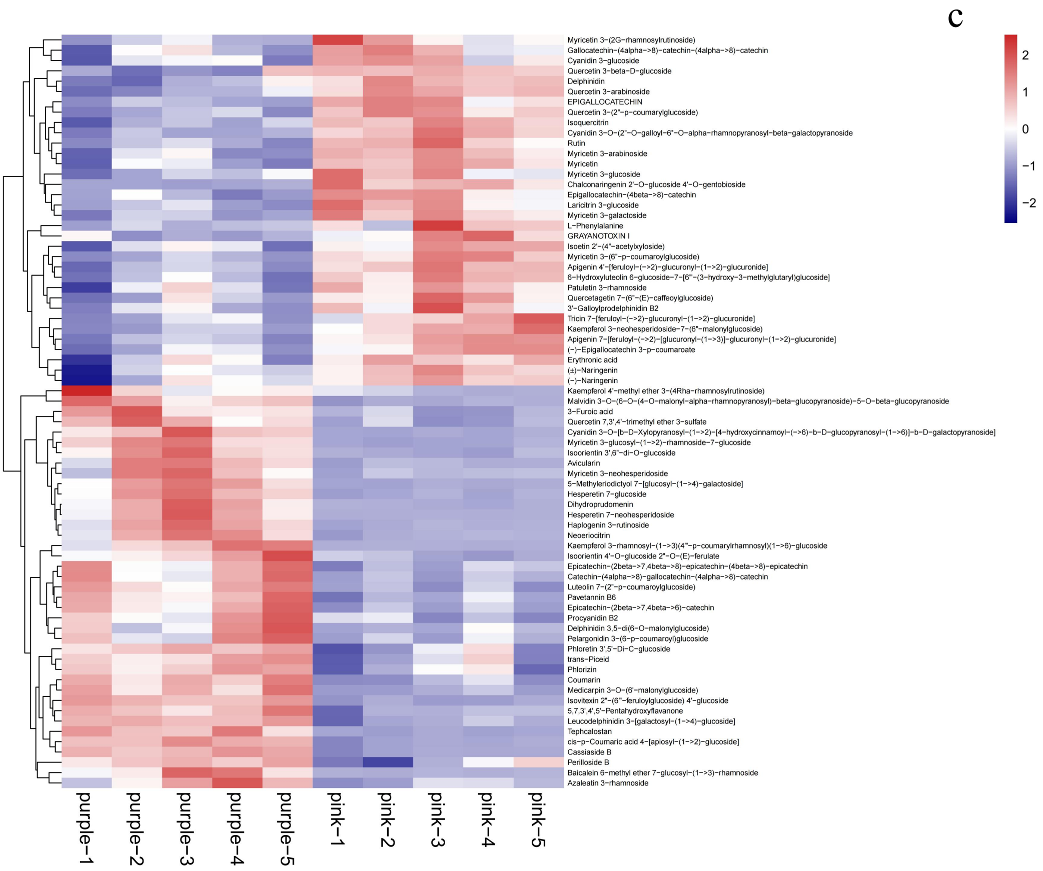

Fig. S2 Hierarchical cluster analysis of differential flavonoid metabolites in *R.pulchrum* Sweet. Note: White, cultivar ‘Baihe’; Pink, cultivar ‘Fenhe’; Purple, cultivar ‘Zihe’. (a)Analysis of flavonoid metabolites between cultivars ‘Baihe’ and ‘Fenhe’;(b)Analysis of flavonoid metabolites between cultivars ‘Zihe’ and ‘Baihe’; (c)Analysis of flavonoid metabolites between cultivars ‘Fenhe’ and ‘Zihe’.
