# Supplementary material for: Integrative analysis of transcriptome and metabolome reveals flavonoid biosynthesis regulation in Rhododendron pulchrum petals
Source: BMC Plant Biol. 2022 Aug 16;22:401. doi: 10.1186/s12870-022-03762-y (PMC9380304; doi:10.1186/s12870-022-03762-y)
Supplement: Supplementary file 3 — Additional file 3: Fig. S3. Transcription factor distribution between three R.pulchrum Sweet cultivars. Note: BMJ, cultivar ‘Baihe’; FMJ, cultivar ‘Fenhe’; ZMJ, cultivar ‘Zihe’. (a) Transcription factor distribution between cultivars ‘Baihe’ and ‘Fenhe’; (b) Transcription factor distribution between cultivars ‘Zihe’ and ‘Baihe’; (c) Transcription factor distribution between cultivars ‘Fenhe’ and ‘Zihe’. [file 12870_2022_3762_MOESM3_ESM.pdf]

a

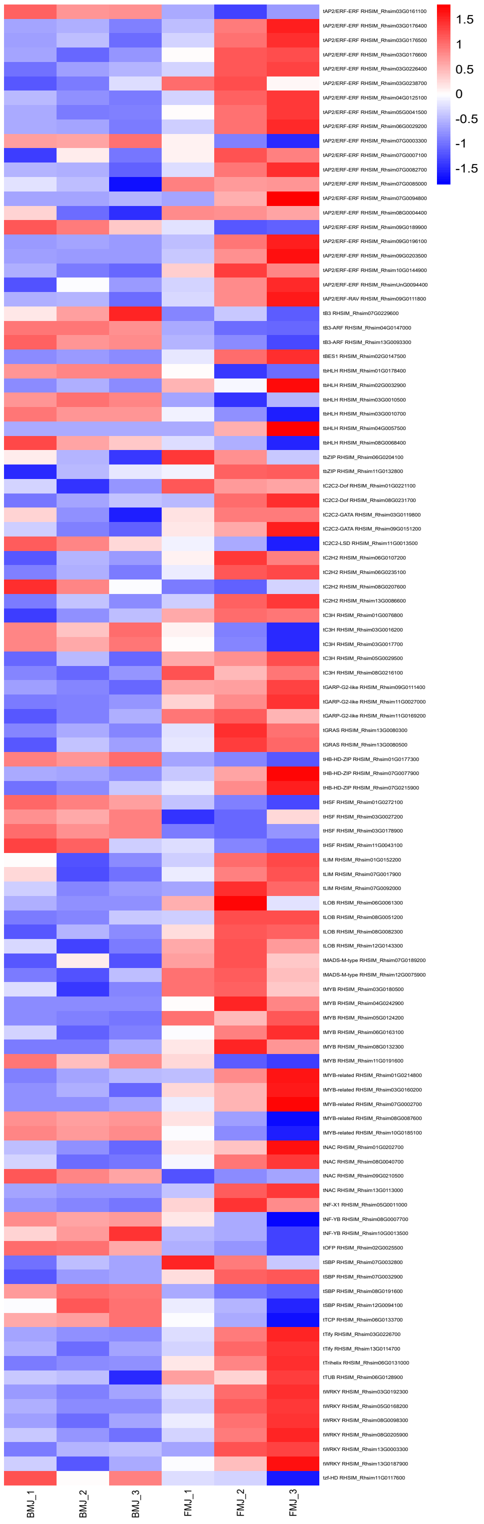
tAP2/ERF-ERF RHSIM_Rhsim03G0161100
tAP2/ERF-ERF RHSIM_Rhsim03G0176400
tAP2/ERF-ERF RHSIM_Rhsim03G0176500
tAP2/ERF-ERF RHSIM_Rhsim03G0176600
tAP2/ERF-ERF RHSIM_Rhsim03G0226400
tAP2/ERF-ERF RHSIM_Rhsim03G0238700
tAP2/ERF-ERF RHSIM_Rhsim04G0125100
tAP2/ERF-ERF RHSIM_Rhsim05G0041500
tAP2/ERF-ERF RHSIM_Rhsim06G0029200
tAP2/ERF-ERF RHSIM_Rhsim07G0003300
tAP2/ERF-ERF RHSIM_Rhsim07G0007100
tAP2/ERF-ERF RHSIM_Rhsim07G0082700
tAP2/ERF-ERF RHSIM_Rhsim07G0085000
tAP2/ERF-ERF RHSIM_Rhsim07G0094800
tAP2/ERF-ERF RHSIM_Rhsim08G0004400
tAP2/ERF-ERF RHSIM_Rhsim09G0189900
tAP2/ERF-ERF RHSIM_Rhsim09G0196100
tAP2/ERF-ERF RHSIM_Rhsim09G0203500
tAP2/ERF-ERF RHSIM_Rhsim10G0144900
tAP2/ERF-ERF RHSIM_RhsimUnG0094400
tAP2/ERF-RAV RHSIM_Rhsim09G0111800
tB3 RHSIM_Rhsim07G0229600
tB3-ARF RHSIM_Rhsim04G0147000
tB3-ARF RHSIM_Rhsim13G0093300
tBES1 RHSIM_Rhsim02G0147500
tbHLH RHSIM_Rhsim01G0178400
tbHLH RHSIM_Rhsim02G0032900
tbHLH RHSIM_Rhsim03G0010500
tbHLH RHSIM_Rhsim03G0010700
tbHLH RHSIM_Rhsim04G0057500
tbHLH RHSIM_Rhsim08G0068400
tbZIP RHSIM_Rhsim06G0204100
tbZIP RHSIM_Rhsim11G0132800
tC2C2-Dof RHSIM_Rhsim01G0221100
tC2C2-Dof RHSIM_Rhsim08G0231700
tC2C2-GATA RHSIM_Rhsim03G0119800
tC2C2-GATA RHSIM_Rhsim09G0151200
tC2C2-LSD RHSIM_Rhsim11G0013500
tC2H2 RHSIM_Rhsim06G0107200
tC2H2 RHSIM_Rhsim06G0235100
tC2H2 RHSIM_Rhsim08G0207600
tC2H2 RHSIM_Rhsim13G0086600
tC3H RHSIM_Rhsim01G0076800
tC3H RHSIM_Rhsim03G0016200
tC3H RHSIM_Rhsim03G0017700
tC3H RHSIM_Rhsim05G0029500
tC3H RHSIM_Rhsim08G0216100
tGARP-G2-like RHSIM_Rhsim09G0111400
tGARP-G2-like RHSIM_Rhsim11G0027000
tGARP-G2-like RHSIM_Rhsim11G0169200
tGRAS RHSIM_Rhsim13G0080300
tGRAS RHSIM_Rhsim13G0080500
tHB-HD-ZIP RHSIM_Rhsim01G0177300
tHB-HD-ZIP RHSIM_Rhsim07G0077900
tHB-HD-ZIP RHSIM_Rhsim07G0215900
tHSF RHSIM_Rhsim01G0272100
tHSF RHSIM_Rhsim03G0027200
tHSF RHSIM_Rhsim03G0178900
tHSF RHSIM_Rhsim11G0043100
tLIM RHSIM_Rhsim01G0152200
tLIM RHSIM_Rhsim07G0017900
tLIM RHSIM_Rhsim07G0092000
tLOB RHSIM_Rhsim06G0061300
tLOB RHSIM_Rhsim08G0051200
tLOB RHSIM_Rhsim08G0082300
tLOB RHSIM_Rhsim12G0143300
tMADS-M-type RHSIM_Rhsim07G0189200
tMADS-M-type RHSIM_Rhsim12G0075900
tMYB RHSIM_Rhsim03G0180500
tMYB RHSIM_Rhsim04G0242900
tMYB RHSIM_Rhsim05G0124200
tMYB RHSIM_Rhsim06G0163100
tMYB RHSIM_Rhsim08G0132300
tMYB RHSIM_Rhsim11G0191600
tMYB-related RHSIM_Rhsim01G0214800
tMYB-related RHSIM_Rhsim03G0160200
tMYB-related RHSIM_Rhsim07G0002700
tMYB-related RHSIM_Rhsim08G0087600
tMYB-related RHSIM_Rhsim10G0185100
tNAC RHSIM_Rhsim01G0202700
tNAC RHSIM_Rhsim08G0040700
tNAC RHSIM_Rhsim09G0210500
tNAC RHSIM_Rhsim13G0113000
tNF-X1 RHSIM_Rhsim05G0011000
tNF-YB RHSIM_Rhsim08G0007700
tNF-YB RHSIM_Rhsim10G0013500
tOFP RHSIM_Rhsim02G0025500
tSBP RHSIM_Rhsim07G0032800
tSBP RHSIM_Rhsim07G0032900
tSBP RHSIM_Rhsim08G0191600
tSBP RHSIM_Rhsim12G0094100
tTCP RHSIM_Rhsim06G0133700
tTify RHSIM_Rhsim03G0226700
tTify RHSIM_Rhsim13G0114700
tTrihelix RHSIM_Rhsim06G0131000
tTUB RHSIM_Rhsim06G0128900
tWRKY RHSIM_Rhsim03G0192300
tWRKY RHSIM_Rhsim05G0168200
tWRKY RHSIM_Rhsim08G0098300
tWRKY RHSIM_Rhsim08G0205900
tWRKY RHSIM_Rhsim13G0003300
tWRKY RHSIM_Rhsim13G0187900
tzf-HD RHSIM_Rhsim11G0117600
BMJ_1
BMJ_2
BMJ_3
FMJ_1
FMJ_2
FMJ_3
1.5
1
0.5
0
-0.5
-1
-1.5

b

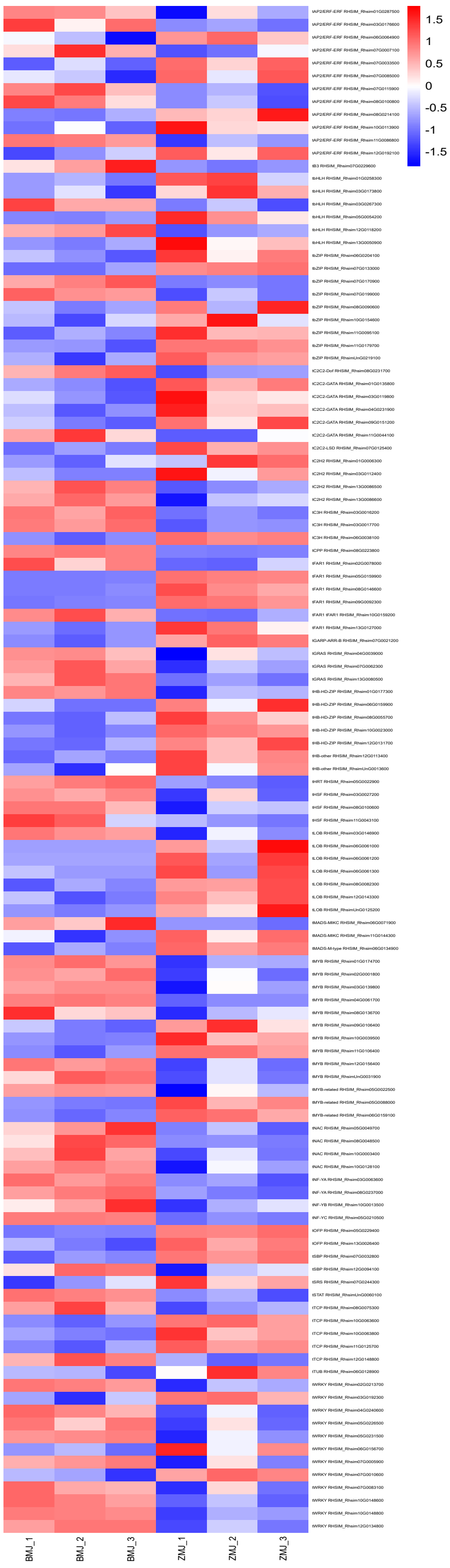

c

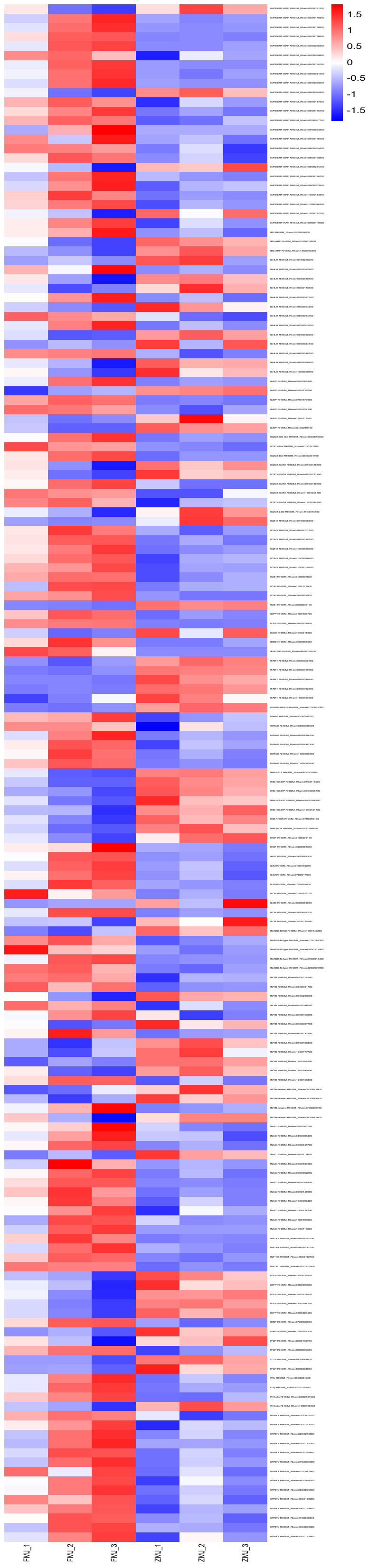

Fig.S3 Transcription factor distribution between three *R.pulchrum* Sweet cultivars. Note: BMJ, cultivar ‘Baihe’; FMJ, cultivar ‘Fenhe’; ZMJ, cultivar ‘Zihe’.(a) Transcription factor distribution between cultivars ‘Baihe’ and ‘Fenhe’;(b) Transcription factor distribution between cultivars ‘Zihe’ and ‘Baihe’;(c) Transcription factor distribution between cultivars ‘Fenhe’ and ‘Zihe’.
