# Supplementary material for: Integrative analysis of transcriptome and metabolome reveals flavonoid biosynthesis regulation in Rhododendron pulchrum petals
Source: BMC Plant Biol. 2022 Aug 16;22:401. doi: 10.1186/s12870-022-03762-y (PMC9380304; doi:10.1186/s12870-022-03762-y)
Supplement: Supplementary file 4 — Additional file 4: Fig. S4. Transcript accumulation measurements of colour-related genes involved in the flavonoid metabolic process. Note: BMJ, cultivar ‘Baihe’; FMJ, cultivar ‘Fenhe’; ZMJ, cultiv. [file 12870_2022_3762_MOESM4_ESM.pdf]

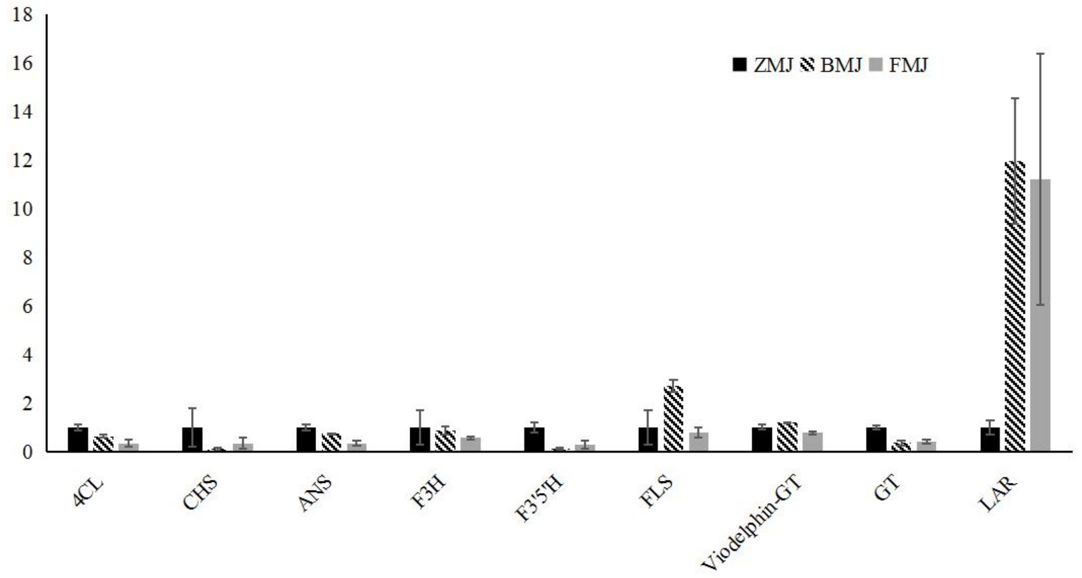

Fig.S4 Transcript accumulation measurements of colour-related genes involved in the flavonoid metabolic process.

Note: BMJ, cultivar 'Baihe'; FMJ, cultivar 'Fenhe'; ZMJ, cultivar 'Zihe'.
