# Supplementary material for: Integrative analysis of transcriptome and metabolome reveals flavonoid biosynthesis regulation in Rhododendron pulchrum petals
Source: BMC Plant Biol. 2022 Aug 16;22:401. doi: 10.1186/s12870-022-03762-y (PMC9380304; doi:10.1186/s12870-022-03762-y)
Supplement: Supplementary file 6 — Additional file 6: Table S2. The anthocyanin derivatives between three comparison groups of R.pulchrum Sweet. [file 12870_2022_3762_MOESM6_ESM.pdf]

Table S2 The anthocyanin derivatives between three comparison groups of *R.pulchrum* Sweet

Group	Anthocyanin	log2(FC)
'Baihe' Vs 'Feneh'	Leucodelphinidin 3-[galactosyl-(1->4)-glucoside]	3.49
	Malvidin	-0.58
'Zihe' Vs 'Baihe'	Malvidin 3-O-(6-O-(4-O-malonyl-alpha-rhamnopyranosyl)-beta-glucopyranoside)-5-O-beta-glucopyranoside	9.64
	Cyanidin 3-O-[b-D-Xylopyranosyl-(1->2)-[4-hydroxycinnamoyl-(->6)-b-D-glucopyranosyl-(1->6)]-b-D-galactopyranoside]	7.08
	Leucodelphinidin 3-[galactosyl-(1->4)-glucoside]	4.99
	Delphinidin	-0.41
	Cyanidin 3-O-(2''-O-galloyl-6''-O-alpha-rhamnopyranosyl-beta-galactopyranoside	-1.68
'Fenhe' Vs 'Zihe'	Cyanidin 3-O-[b-D-Xylopyranosyl-(1->2)-[4-hydroxycinnamoyl-(->6)-b-D-glucopyranosyl-(1->6)]-b-D-galactopyranoside]	4.27
	Malvidin 3-O-(6-O-(4-O-malonyl-alpha-rhamnopyranosyl)-beta-glucopyranoside)-5-O-beta-glucopyranoside	3.18
	Leucodelphinidin 3-[galactosyl-(1->4)-glucoside]	1.50
	Pelargonidin 3-(6-p-coumaroyl)glucoside	0.93
	Delphinidin 3,5-di(6-O-malonylglucoside)	0.71
	Delphinidin	-0.51
	Cyanidin 3-O-(2''-O-galloyl-6''-O-alpha-rhamnopyranosyl-beta-galactopyranoside	-1.90
