# Supplementary material for: Integrative analysis of transcriptome and metabolome reveals flavonoid biosynthesis regulation in Rhododendron pulchrum petals
Source: BMC Plant Biol. 2022 Aug 16;22:401. doi: 10.1186/s12870-022-03762-y (PMC9380304; doi:10.1186/s12870-022-03762-y)
Supplement: Supplementary file 7 — Additional file 7: Table S3. Transcriptome sequencing results from three R.pulchrum Sweet cultivars. [file 12870_2022_3762_MOESM7_ESM.pdf]

Table S3 Transcriptome sequencing results from three *R.pulchrum* Sweet cultivars

Sample	RawReads	RawBases	CleanReads	CleanBases	ValidBases	Q30	GC
‘Baihe’-1	47.84M	7.18G	47.02M	6.61G	92.18%	93.91%	46.87%
‘Baihe’-2	49.30M	7.40G	48.46M	6.87G	92.84%	94.36%	46.82%
‘Baihe’-3	49.80M	7.40G	48.99M	6.96G	93.12%	94.36%	46.80%
‘Fenhe’-1	51.67M	7.75G	50.83M	7.19G	92.78%	94.30%	46.92%
‘Fenhe’-2	50.35M	7.55G	49.54M	7.06G	93.45%	94.30%	47.00%
‘Fenhe’-3	48.87M	7.33G	48.09M	6.80G	92.81%	94.42%	46.91%
‘Zihe’-1	47.68M	7.15G	46.92M	6.62G	92.56%	94.11%	47.18%
‘Zihe’-2	51.55M	7.73G	50.66M	7.16G	92.65%	94.04%	46.85%
‘Zihe’-3	47.35M	7.10G	46.51M	6.57G	92.45%	94.03%	47.19%
