# Supplementary material for: Integrative analysis of transcriptome and metabolome reveals flavonoid biosynthesis regulation in Rhododendron pulchrum petals
Source: BMC Plant Biol. 2022 Aug 16;22:401. doi: 10.1186/s12870-022-03762-y (PMC9380304; doi:10.1186/s12870-022-03762-y)
Supplement: Supplementary file 10 — Additional file 10: Table S6. The primers used for qRT-PCR analysis. [file 12870_2022_3762_MOESM10_ESM.pdf]

Table S6 The primers used for qRT-PCR analysis

Primer ID	Gene ID	Forward primer	Reverse primer
4CL	RHSIM_Rhsim10G0185800	AAGCCAAGGTCTTCTTTGT	CTTCCAACTCGGGCATTAG
CHS	RHSIM_Rhsim09G0036400	GTGTGTTCTGAGATCACGG	GAACATGGCCTGGCTAACT
ANS	RHSIM_Rhsim07G0096600	CCTAACCACCAAGGTCCTAT	ATCTGGATGAGGAGTTCGT
F3H	RHSIM_Rhsim11G0169400	AGTTCTTTGCACAGACGAC	CCTGACATTCTTGGTATGCTC
F3'5'H	RHSIM_Rhsim13G0208100	GATGCTGACGTTCGCTAT	TCCACCACCATGTCCTTG
FLS	RHSIM_Rhsim03G0144500	AAATTCACCGAGGACTTACG	AGGAAATAATAGTCGCTCCAG
Viodelphin-GT	RHSIM_RhsimUnG0183300	TGGAAGGTTGGCCTTCAT	AGAAATTGAGGGTGTTCGAC
GT	RHSIM_Rhsim06G0140700	GACCAGACGGAAATGCTC	ATGCGGACGTACTCTTTG
LAR	RHSIM_Rhsim12G0144700	GGACAGAGGCGCTATAGT	ACCCACCGCTGATATTACCA
GAPDH		TCGGAATCAACGGTTTTGGAA	CACTTGACCGTGAACACTGT
